# Extracellular C1qbp inhibits myogenesis by suppressing NFATc1

**DOI:** 10.1038/s41598-024-66549-1

**Published:** 2024-07-08

**Authors:** Jin-Man Kim, Ho Kyoung Kim, Han Jin Cho, Sung-Ah Moon, Yewon Kim, Jeong Yeon Hong, Seung Hun Lee, Kyunggon Kim, Jung-Min Koh

**Affiliations:** 1 Asan Institute for Life Sciences, Asan Medical Center, Seoul, 05505 Republic of Korea; 2 AMIST, Asan Medical Center, University of Ulsan College of Medicine, Seoul, 05505 Republic of Korea; 3 Department of Biomedical Science, University of Ulsan College of Medicine, Seoul, 05505 Republic of Korea; 4 Division of Endocrinology and Metabolism, Asan Medical Center, University of Ulsan College of Medicine, Seoul, 05505 Republic of Korea; 5 Department of Convergence Medicine, Asan Institute for Life Sciences, Asan Medical Center, University of Ulsan College of Medicine, Seoul, 05505 Republic of Korea

**Keywords:** C1qbp, Myogenesis, NFATc1, p300, Exercise, Hindlimb unloading, Cell biology, Molecular biology, Ageing, Differentiation

## Abstract

Aging and lack of exercise are the most important etiological factors for muscle loss. We hypothesized that new factors that contribute to muscle loss could be identified from ones commonly altered in expression in aged and exercise-limited skeletal muscles. Mouse gastrocnemius muscles were subjected to mass spectrometry-based proteomic analysis. The muscle proteomes of hindlimb-unloaded and aged mice were compared to those of exercised and young mice, respectively. C1qbp expression was significantly upregulated in the muscles of both hindlimb-unloaded and aged mice. In vitro myogenic differentiation was not affected by altering intracellular C1qbp expression but was significantly suppressed upon recombinant C1qbp treatment. Additionally, recombinant C1qbp repressed the protein level but not the mRNA level of NFATc1. NFATc1 recruited the transcriptional coactivator p300, leading to the upregulation of acetylated histone H3 levels. Furthermore, NFATc1 silencing inhibited p300 recruitment, downregulated acetylated histone H3 levels, and consequently suppressed myogenic differentiation. The expression of C1qbp was inversely correlated with that of NFATc1 in the gastrocnemius muscles of exercised or hindlimb-unloaded, and young or aged mice. These findings demonstrate a novel role of extracellular C1qbp in suppressing myogenesis by inhibiting the NFATc1/p300 complex. Thus, C1qbp can serve as a novel therapeutic target for muscle loss.

## Introduction

Sarcopenia is characterized by a progressive and general loss of skeletal muscle and strength^1^. The aging population is rapidly increasing worldwide. Thus, sarcopenia is a major health challenge in aging societies^2^. Its traditional therapeutic targets, such as myostatin/activin receptor and androgen receptor, have been aimed to develop several drugs in preclinical and clinical trials, but no drugs have yet been approved for sarcopenia due to insufficient therapeutic efficacy and/or occurrence of side effects^2^. Therefore, other novel therapeutic targets for sarcopenia needed to be discovered.

Aging and lack of exercise are the most important etiological factors for sarcopenia^2^. Mechanistically, these processes are quite different in that the former primarily affects type II and the latter type I muscle fibers^3,4^. Type I fibers are more fatigue resistant, while type II fibers are associated with power generation^5^. Since improvement in both fatigue resistance and muscle strength may be necessary to overcome sarcopenia, factors acting on both fiber types are likely to be more appropriate therapeutic targets for sarcopenia. Therefore, factors influenced by both aging and lack of exercise may be more appropriate potential therapeutic targets for sarcopenia. This study used non-targeted proteomic analysis to explore factors altered in the skeletal muscles of aged and hindlimb-unloaded mice.

On the other hand, recent improvements in antibody development technologies and the discovery of many suitable ligands have greatly changed the landscape of antibody-based drug discovery^6^. Monoclonal antibodies as therapeutics have several advantages including high drug selectivity and pharmacokinetic benefits^6^. Therefore, we wanted to identify factors that might be suitable ligands for blocking antibody drug development, and specified several potential conditions for this purpose. First, they must induce muscle atrophy extracellularly. Second, they must be upregulated in atrophied muscle. Finally, it should be a novel therapeutic target for sarcopenia. This study demonstrated that extracellular C1qbp is a novel therapeutic target for sarcopenia that meets these conditions.

## Results

### Proteomic analysis revealed that C1qbp is a novel anti-myogenic factor

**Figure 1:**
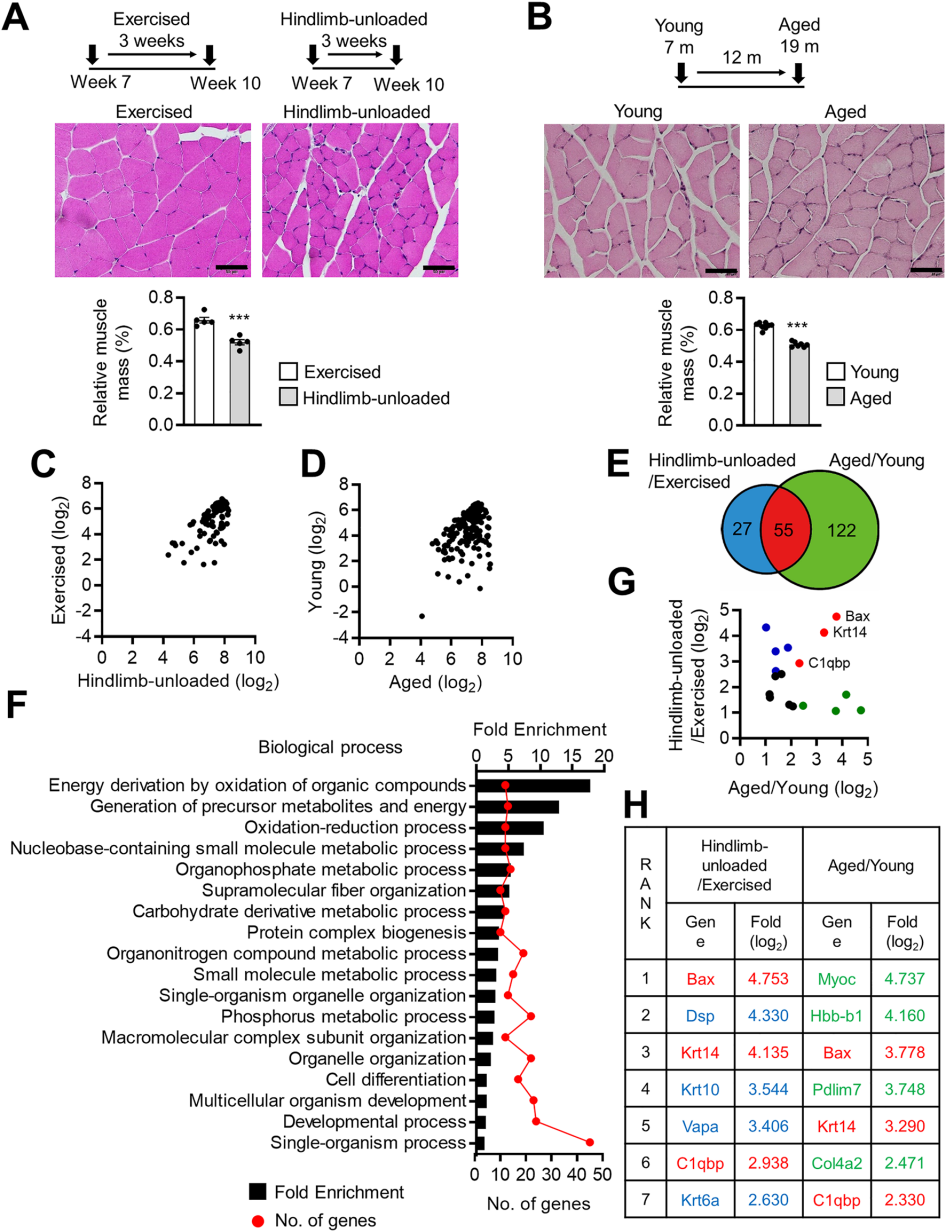
Muscle proteome of mice with muscle loss. (**A**) To establish the exercise model, male C57BL/6 mice aged 7 weeks were exercised on a treadmill for 30 min a day for 3 weeks. To establish the hindlimb-unloaded model, the tail of male C57BL/6 mice aged 7 weeks was suspended for 3 weeks. Gastrocnemius muscles isolated from exercised and hindlimb-unloaded mice were fixed, embedded, sectioned, and stained with hematoxylin and eosin (H&E) for histological analysis. Representative images of H&E-stained sections are shown in the middle panels. Scale bar, 50 μm. Quantification of relative muscle mass is shown in the lower. Data are represented as mean ± SEM (n = 5). (**B**) Gastrocnemius muscles were isolated from 7-month-old (young group) or 19-month-old (aged group) male C57BL/6 mice. Representative images of H&E-stained sections and quantification of relative muscle mass are shown in the middle and lower, respectively. Scale bar, 50 μm. Data are represented as mean ± SEM (Young = 8, Aged = 7). (**C**) A Scatter plot comparing fold change between hindlimb-unloaded and exercised mice is shown. Compared with those in the exercised mice, the muscle expression levels of 82 proteins were significantly upregulated in hindlimb-unloaded mice. (**D**) A Scatter plot comparing fold change between aged and young mice is shown. Compared with those in young mice, the muscle expression levels of 177 proteins were significantly upregulated in aged mice. (**E**) Venn diagrams represent 55 proteins that were upregulated more than two-fold in hindlimb-unloaded and aged mice compared to exercised and young mice, respectively. (**F**) Gene ontology enrichment analysis of the 55 common target proteins using the DAVID bioinformatics database (https://david.ncifcrf.gov). (**G**) A scatter plot comparing fold change between aged/young groups and hindlimb-unloaded/exercised groups is shown. The graph includes 17 cell differentiation-related proteins. Upregulated proteins in the hindlimb-unloaded, aged, and both groups are marked in blue, green, and red, respectively. (**H**) Cell differentiation-related proteins are ranked in the order of fold change in both hindlimb-unloaded vs. exercised and aged vs. young groups. The top seven ranked proteins in both hindlimb-unloaded vs. exercised and aged vs. young are shown. Upregulated proteins in hindlimb-unloaded, aged, and both groups are marked in blue, green, and red, respectively. ***P < 0.001.

Two mouse models with muscle loss (hindlimb-unloaded and aged mice) were generated. The proteomes of hindlimb-unloaded and aged mice were compared with those of exercised and young mice, respectively^7,8^. The cross-sectional areas of the muscles of hindlimb-unloaded and aged mice were lower than those of exercised and young mice, respectively, which confirmed that the two mouse models with muscle loss exhibited muscle atrophy (Fig. 1A,B). The gastrocnemius muscles of these mice were subjected to mass spectrometry-based proteomic analysis. Compared with those in exercised and young mice, the muscle expression levels of 82 and 177 proteins were significantly upregulated in hindlimb-unloaded (Fig. 1C and Supplementary Table S1) and aged mice (Fig. 1D and Supplementary Table S2), respectively. Of these, 55 proteins were upregulated in both hindlimb-unloaded and aged mice (Fig. 1E). Gene ontology analysis revealed that these proteins were involved in several biological process categories, such as energy production via the oxidation of organic compounds, metabolic processes of various substances, and cell differentiation (Fig. 1F). Among the factors involved in energy production, no new candidate factors were found that met the criteria described in the Introduction. Therefore, this study focused on the proteins involved in cell differentiation, which is critical in controlling and maintaining the growth and development of skeletal muscle^9,10^. The top three proteins upregulated in the gastrocnemius muscles of hindlimb-unloaded and aged mice were Bax, Krt14, and C1qbp (Fig. 1G,H).

**Figure 2:**
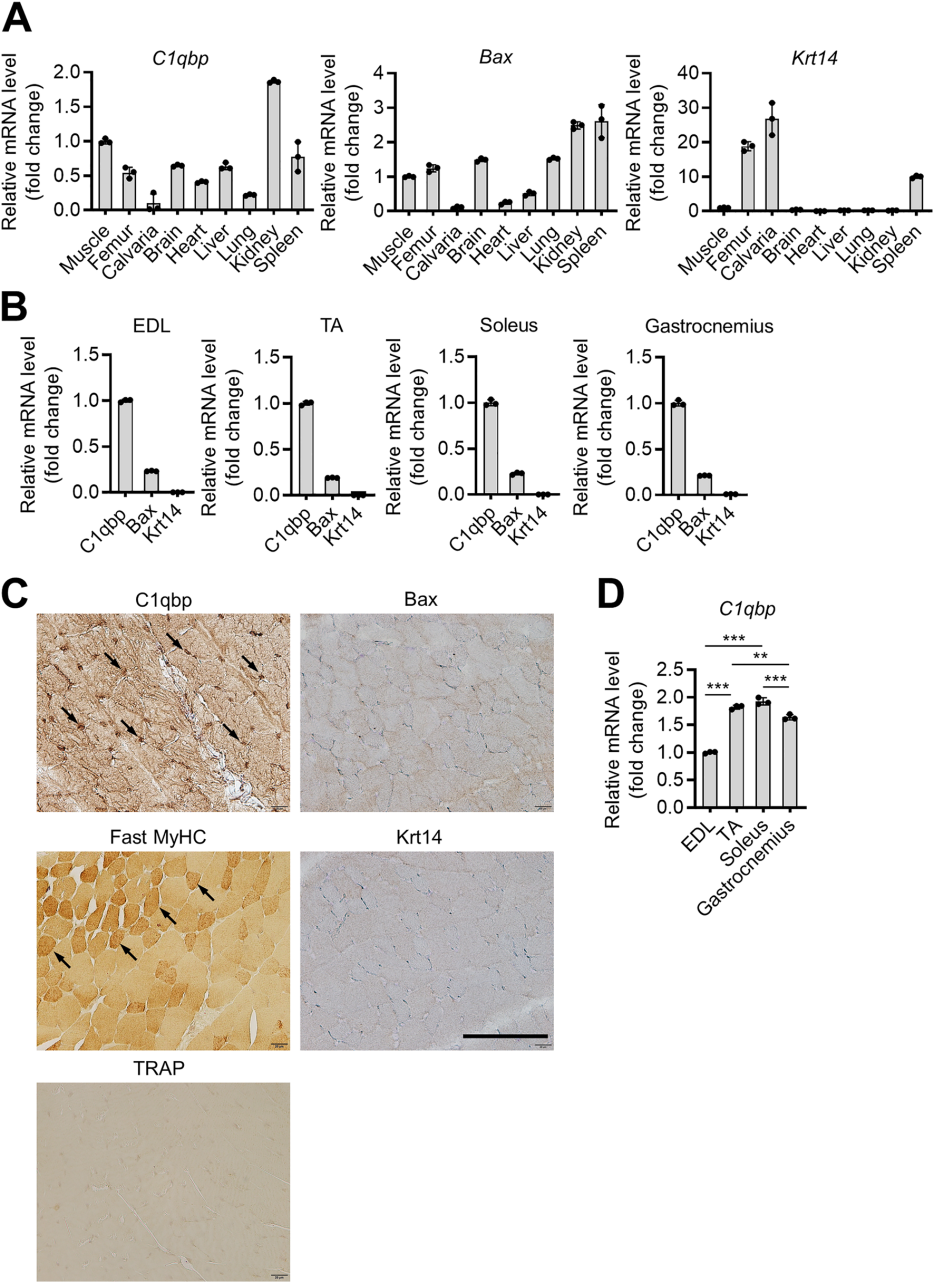
C1qbp expression in skeletal muscles. (**A**) Total RNAs were isolated from various tissues of male C57BL/6 mice aged 8 weeks. The expression levels of *C1qbp*, *Krt14*, and *Bax* were quantified using qRT-PCR. Muscle indicates gastrocnemius. Data are represented as mean ± SD (n = 3). (**B**) The expression levels of *C1qbp*, *Krt14*, and *Bax* in various skeletal muscles were examined using qRT-PCR. *EDL* extensor digitorum longus, *TA* tibialis anterior. Data are represented as mean ± SD (n = 3). (**C**) Gastrocnemius muscles of male C57BL/6 mice aged 8 weeks were subjected to immunohistochemical analysis with anti-C1qbp, anti-Krt14, and anti-Bax antibodies. Anti-MyHC and anti-tartrate-resistant acid phosphatase (TRAP) antibodies were used as positive and negative controls, respectively. Representative immunohistochemical images are shown. Scale bar, 100 μm. Arrows indicate the positive signals. (**D**) The expression level of *C1qbp* in various skeletal muscle types was analyzed by qRT-PCR. *EDL* extensor digitorum longus, *TA* tibialis anterior. Data are represented as mean ± SD (n = 3). **P < 0.01; ***P < 0.001.

The expression levels of *Bax*, *Krt14*, and *C1qbp* in various tissues were determined using quantitative real-time polymerase chain reaction (qRT-PCR). The kidney exhibited the highest *C1qbp* expression, followed by muscles (Fig. 2A). However, the expression of *Bax* in muscles was lower than that in other tissues. The expression of *Krt14* was not detected in muscles (Fig. 2A). Comparative analysis of the expression levels of *C1qbp* in various muscle tissues revealed that the expression of *C1qbp* was the highest in all tested muscles than that of *Bax* and *Krt14* (Fig. 2B). This was further confirmed by subjecting gastrocnemius muscles to immunohistochemical staining (Fig. 2C). qRT-PCR showed that *C1qbp* was most highly expressed in the soleus and tibialis anterior among the muscles tested (Fig. 2D). Thus, this study further examined the role of C1qbp in myogenic differentiation.

**Figure 3:**
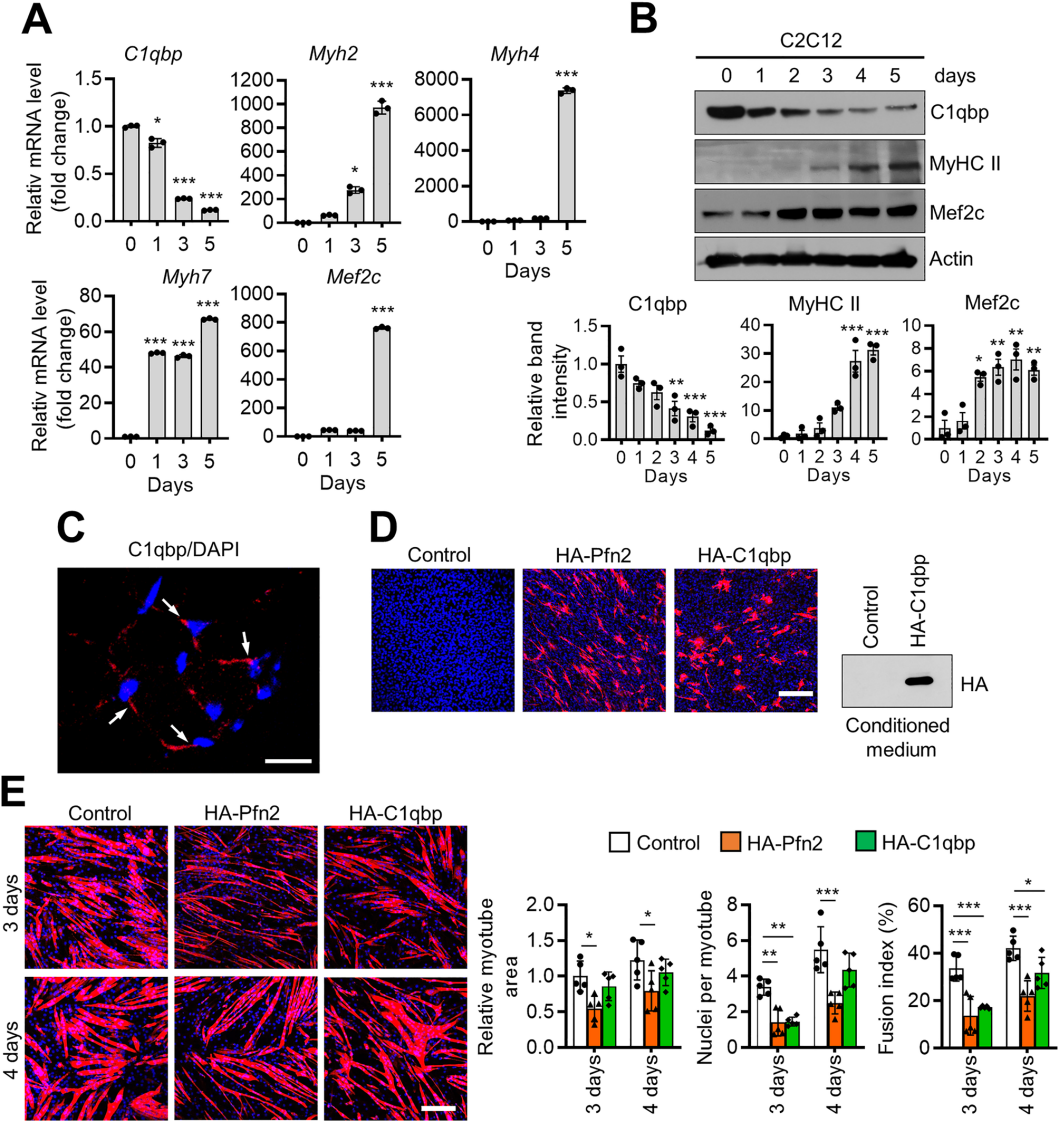
C1qbp expression in muscle cells. (**A**) C2C12 cells were differentiated using a culture medium containing 2% horse serum for the indicated times. The expression levels of *C1qbp*, *Myh2*, *Myh4*, *Myh7*, and *Mef2c* were determined using qRT-PCR. Data are represented as mean ± SD (n = 3). (**B**) Lysates were prepared from C2C12 cells after differentiation with 2% horse serum for the indicated durations. The expression levels of C1qbp, MyHC II, and Mef2c were determined using western blotting. A representative band is shown, and their relative band densities are shown in the lower. Data are represented as mean ± SEM (n = 3). (**C**) Gastrocnemius muscles were immunostained with anti-C1qbp (red) antibody, while the nuclei were stained with DAPI (blue). A representative image is shown. White arrows indicate C1qbp-positive cells. Scale bar, 20 μm. (**D**) C2C12 cells were transfected with mock (control) or hemagglutinin (HA)-tagged Pfn2 (HA-Pfn2) and HA-tagged C1qbp (HA-C1qbp) plasmids for 48 h. The transfection efficiencies of HA-Pfn2 and HA-C1qbp were 11.96 ± 2.28% and 9.80 ± 0.66% using immunostaining with anti-HA antibody (red). Nuclei were stained with DAPI (blue). The HA-C1qbp expression in conditioned medium (right) were subjected to western blotting. Scale bar, 200 μm. (**E**) C2C12 cells were transfected with the HA-Pfn2 and HA-C1qbp constructs for 24 h and differentiated into myotubes for 3 and 4 days. The cells were immunostained with anti-MyHC (red) antibody, while the nuclei were stained with DAPI (blue). Representative immunostaining images are shown in the left. The myotube areas, nuclei number per myotube, and fusion index were analyzed using the Image-Pro Plus and Image J programs, and their quantification results are shown in the right. Data are represented as mean ± SD (n = 5). Scale bar, 200 μm. Original blots in (**B**,**D**) are presented in Supplementary Fig. S3. * < 0.05; **P < 0.01; ***P < 0.001.

The expression of C1qbp was determined during myogenic differentiation. In contrast to the upregulated expression patterns of myogenic differentiation markers, the mRNA (Fig. 3A) and protein (Fig. 3B) expression levels of C1qbp were downregulated. Immunofluorescent staining revealed that most C1qbp may be mainly localized to the mitochondria (Fig. 3C). Hence, the effect of C1qbp overexpression (Fig. 3D) on myogenic differentiation was examined. Compared with the positive control group, the transfection efficiency of C1qbp was similar (Fig. 3D). C1qbp overexpression also caused more expression in conditioned medium (CM, Fig. 3D). The overexpression partially inhibited myogenic differentiation in C2C12 cells, showing no change in myotube area but decreased nuclei number and fusion (Fig. 3E).

**Figure 4:**
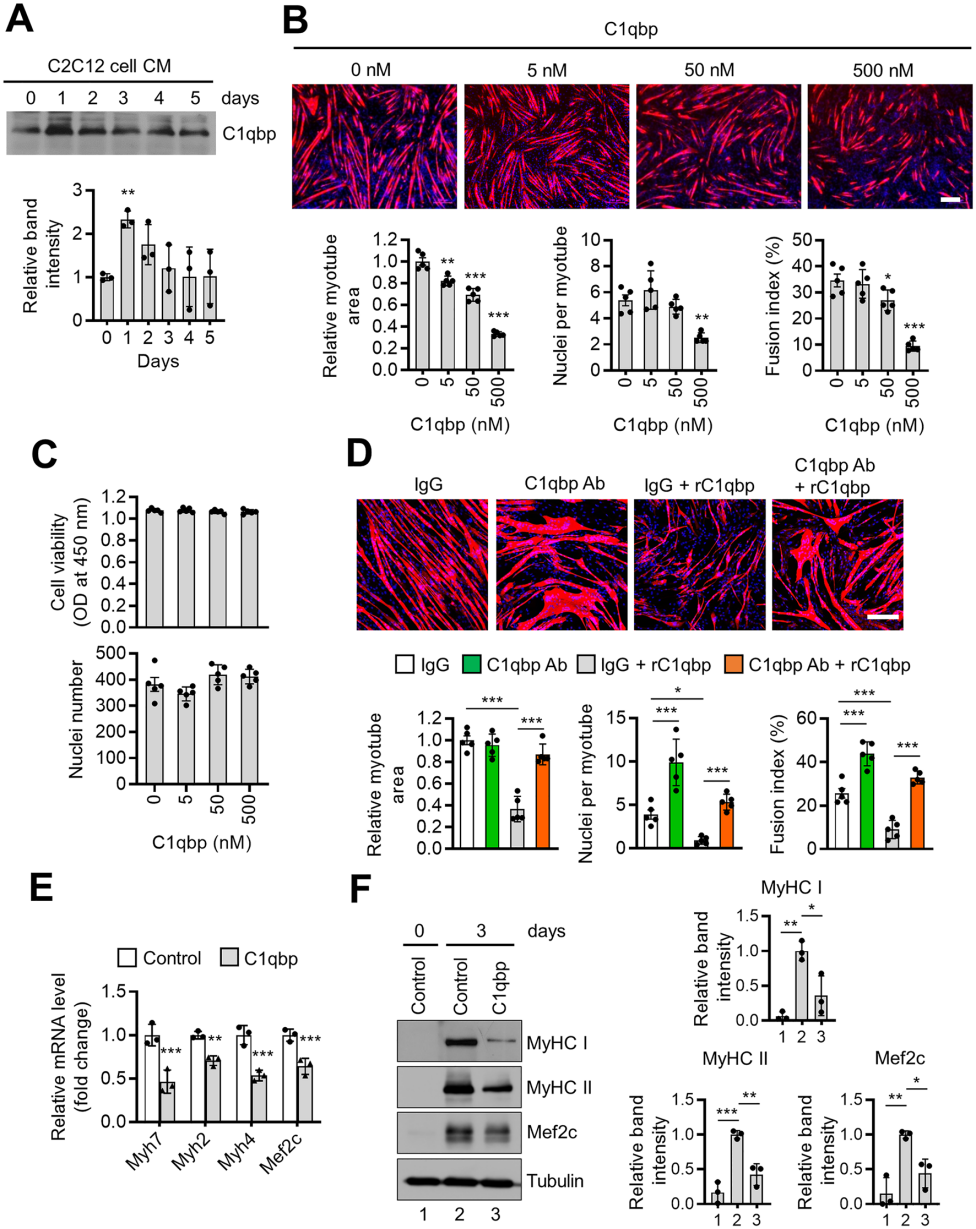
Suppression of myogenic differentiation upon C1qbp treatment. (**A**) C2C12 cells were induced to differentiate with 2% horse serum for the indicated times. The conditioned medium (CM) was harvested. The expression of C1qbp in the CM was analyzed using western blotting. Quantification of relative band density is shown in the lower. Data are represented as mean ± SD (n = 3). (**B**) C2C12 cells were treated with the indicated concentrations of recombinant C1qbp (rC1qbp) and differentiated into myotubes for 3 days. The cells were fixed and immunostained with anti-MyHC (red) antibodies, while the nuclei were stained with DAPI (blue). Representative immunostaining images are shown in the upper panels. The myotube areas, nuclei number per myotube and fusion index were analyzed using the Image-Pro Plus and Image J programs. Data are represented as mean ± SD (n = 5). Scale bar, 200 μm. (**C**) C2C12 cells were treated with rC1qbp for 3 days. Cell viability was measured using the cell counting kit assay. Nuclei number was also determined. Data are represented as mean ± SD (n = 5). (**D**) C2C12 cells were treated with C1qbp antibody (10 μg/ml) and/or rC1qbp (500 nM) and differentiated into myotubes for 4 days. Representative immunostaining images are shown in the upper. Quantification of myotube area, nuclei number per myotube, and fusion index are shown in the lower. Scale bar, 200 μm. Data are represented as mean ± SD (n = 5). (**E**) C2C12 cells were treated with vehicle (control) or 500 nM of rC1qbp and differentiated into myotubes for 3 days. The expression levels of *Myh7*, *Myh2*, *Myh4*, and *Mef2c* were analyzed using qRT-PCR. Data are represented as mean ± SD (n = 3). (**F**) C2C12 cells treated with vehicle (control) or rC1qbp (500 nM) were induced to differentiate for 3 days. The expression levels of MyHC I, MyHC II, and Mef2c were analyzed using western blotting. Tubulin was used as a loading control. Quantification of relative band densities is shown in the right. Data are represented as mean ± SD (n = 3). Original blots in (**A**,**F**) are presented in Supplementary Fig. S4. * < 0.05; *P* < 0.01; ***P < 0.001.

The extracellular form of C1qbp has been previously reported^11–13^. The CM was collected during the myogenic differentiation of C2C12 cells. Western blotting revealed that the expression of C1qbp was upregulated in the CM on day 1 post-induction of differentiation but downregulated with further myogenic differentiation (Fig. 4A). We hypothesized that C1qbp secreted from the muscles may act on myogenic differentiation in an autocrine manner. To test this hypothesis, C2C12 cells were treated with recombinant C1qbp (rC1qbp) proteins. Treatment with rC1qbp dose-dependently suppressed in vitro myogenesis (Fig. 4B) but did not affect the number of myoblasts (Fig. 4C). A neutralizing antibody against C1qbp near completely recovered the rC1qbp-suppressed myogenesis (Fig. 4D), indicating that the inhibitory effect of C1qbp on myogenesis was specific. Consistently, rC1qbp significantly downregulated the expression of myogenic differentiation markers (Fig. 4E,F), indicating that extracellular C1qbp inhibits myogenic differentiation.

### C1qbp suppresses NFATc1 expression

**Figure 5:**
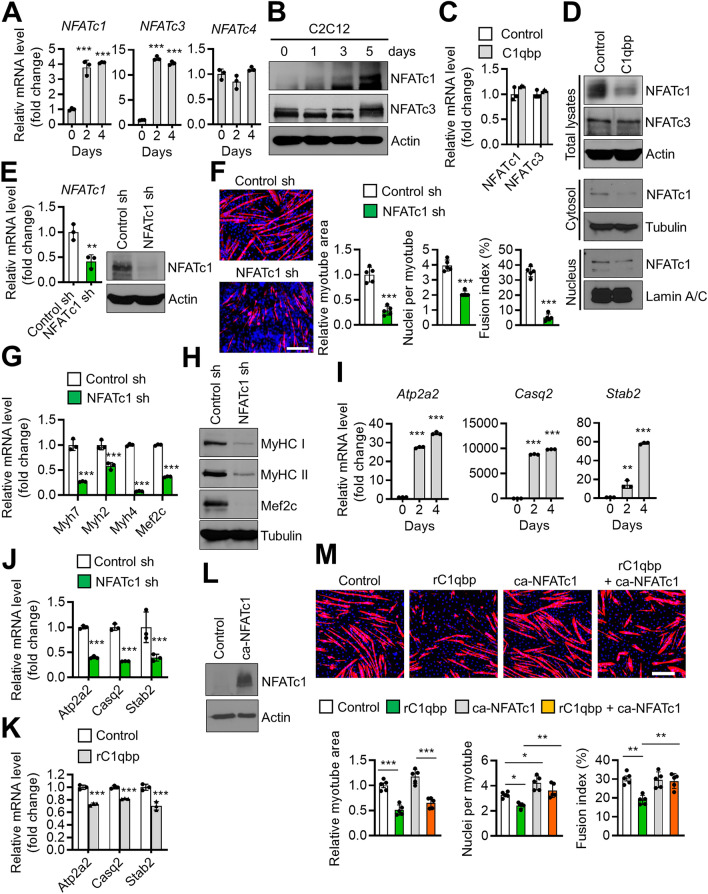
C1qbp-dependent suppression of NFATc1 expression. (**A**,**B**) C2C12 cells were induced to differentiate with 2% horse serum for the indicated times. The expressions of NFAT family were quantified using qRT-PCR and western blotting. Data are represented as mean ± SD (n = 3). (**C**,**D**) C2C12 cells treated with vehicle (control) or 500 nM rC1qbp were induced to differentiate with 2% horse serum for 5 days. The NFATc1 and NFATc3 levels were determined using qRT-PCR (**C**) and western blotting (**D**). The cytosolic (middle, **D**) and nuclear (lower, **D**) fractions were also analyzed by western blotting using anti-NFATc1 antibody. Actin, tubulin and lamin A/C were used as loading controls. Data are represented as mean ± SD (n = 3). (**E**) C2C12 cells were transfected with nontargeting control shRNA (control sh) or NFATc1 shRNA for 48 h. The NFATc1 knockdown efficiency was analyzed using qRT-PCR and western blotting. Data are represented as mean ± SD (n = 3). (**F**) Mock-depleted (control sh) or NFATc1-depleted (NFATc1 sh) C2C12 cells were differentiated into myotubes for 4 days and immunostained with anti-MyHC (red) antibodies, while the nuclei were stained with DAPI (blue). Representative immunostaining images are shown on the left. Quantification of the myotube area, nuclei number per myotube, and fusion index are shown on the right. Data are represented as mean ± SD (n = 5). Scale bar, 200 μm. (**G**,**J**) NFATc1-depleted C2C12 cells were induced to differentiate with a differentiation medium for 3 days. The mRNA levels of the indicated genes were measured using qRT-PCR. Data are represented as mean ± SD (n = 3). (**H**) C2C12 cells were processed as in (**F**). Total cell lysates were subjected to western blotting analysis using the indicated antibodies (loading control: Tubulin). (**I**) C2C12 cells were differentiated with 2% horse serum for 2 and 4 days. The target genes levels were quantified using qRT-PCR. Data are represented as mean ± SD (n = 3). (**K**) C2C12 cells treated with rC1qbp (500 nM) were induced to differentiate with 2% horse serum for 3 days. The expression levels of NFATc1 target genes were analyzed using qRT-PCR. Data are represented as mean ± SD (n = 3). (**L**) C2C12 cells were infected with constitutively active NFATc1 (ca-NFATc1) retrovirus and then cultured for 2 days. The NFATc1 level was analyzed using western blotting. (**M**) ca-NFATc1-overexpressed C2C12 cells were treated rC1qbp (500 nM) and then differentiated into myotubes for 4 days. Representative immunostaining images are shown on the upper. Quantification of the myotube area, nuclei number per myotube, and fusion index are shown on the lower. Data are represented as mean ± SD (n = 5). Scale bar, 200 μm. Original blots in (**B**,**D**,**E**,**H**,**L**) are presented in Supplementary Fig. S5. *P < 0.05; **P < 0.01; ***P < 0.001.

C1qbp is reported to regulate intracellular calcium content^14^, which is closely associated with the nuclear factor of activated T cells (NFAT) family, a transcription factor important for myoblast differentiation^15^. The expression levels of NFATc1 and NFATc3 were upregulated with myogenic differentiation (Fig. 5A,B). However, the expression of *NFATc2* was not detected (data not shown), and that of *NFATc4* did not change during myogenic differentiation (Fig. 5A). Thus, the regulatory effects of C1qbp on the expression of NFATc1 and NFATc3 were examined. Treatment with rC1qbp did not suppress *NFATc1* mRNA level (Fig. 5C), but did suppress both its cytoplasmic and nuclear protein levels (Fig. 5D). Additionally, rC1qbp did not alter NFATc3 expression (Fig. 5C,D).

The activity of NFATc1 in muscle cells was further examined. The knockdown of NFATc1 (Fig. 5E) significantly downregulated myogenic differentiation in C2C12 cells (Fig. 5F). Consistently, myogenic differentiation markers were markedly attenuated in NFATc1 knockdown cells (Fig. 5G,H). Atp2a2, Casq2, and Stab2 are the downstream target genes of NFATc1^16–18^. Similar to those of NFATc1, the expression levels of *Atp2a2*, *Casq2*, and *Stab2* were upregulated with myogenic differentiation (Fig. 5I) but were suppressed upon NFATc1 knockdown (Fig. 5J). This confirmed that Atp2a2, Casq2, and Stab2 are the target genes of NFATc1. Treatment with rC1qbp attenuated the expression of NFATc1 target genes (Fig. 5K). Finally, we tested whether overexpression of NFATc1 may rescue the inhibitory effect of rC1qbp on myogenic differentiation (Fig. 5L). Although its overexpression did not restore the myotube area suppressed by rC1qbp, it did reverse the nuclei number per myotube and the fusion index (Fig. 5M). These findings suggest that C1qbp may inhibit myogenic differentiation partially through the suppression of NFATc1.

### p300 regulates myogenic differentiation by regulating NFATc1 occupancy and histone H3 acetylation

**Figure 6:**
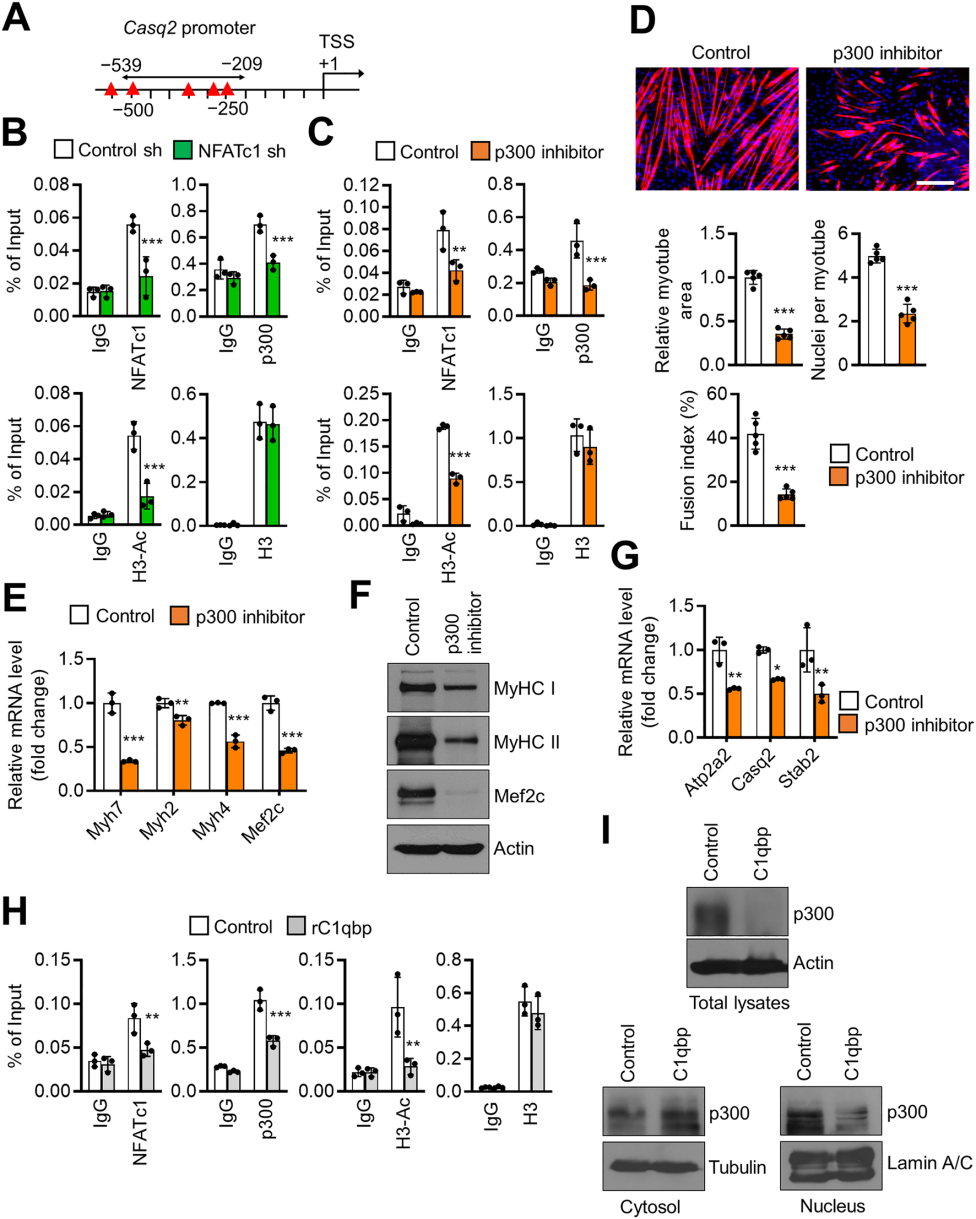
NFATc1-dependent recruitment of p300 at the target genes for myogenic differentiation. (**A**) A scheme of putative NFATc1 binding sites predicted using PROMO (version 3.0.2; http://alggen.lsi.upc.es/cgi-bin/promo_v3/promo/promoinit.cgi?dirDB=TF_8.3) in the 0.55 kb promoter region of *Casq2*. The transcription start site (TSS) is indicated as + 1. The potential NFATc1 binding sites are indicated with red triangles. The location of the primer used for the chromatin immunoprecipitation (ChIP) assay is shown using an arrow. (**B**) Mock-depleted (control sh) or NFATc1-depleted (NFATc1 sh) C2C12 cells were differentiated with 2% horse serum for 5 days. ChIP assays were performed using anti-NFATc1, anti-p300, anti-acetylated histone H3 (H3-Ac), and anti-histone H3 (H3) antibodies. Percent input was determined as the amount of immunoprecipitated DNA relative to input DNA. Data are represented as mean ± SD (n = 3). (**C**) C2C12 cells treated with vehicle (control) or p300 inhibitor (20 μM) were differentiated in the presence of 2% horse serum for 5 days. ChIP assays were performed as described in (**B**). Data are represented as mean ± SD (n = 3). (**D**) Vehicle (control)-treated or p300 inhibitor (20 μM)-treated C2C12 cells were differentiated in the presence of 2% horse serum for 4 days. The cells were stained with anti-MyHC (red) antibodies, while the nuclei were stained with DAPI (blue). Quantification of the myotube area, nuclei number per myotube, and fusion index are shown on the lower. Scale bar, 200 μm. Data are represented as mean ± SD (n = 5). (**E**,**G**) C2C12 cells treated with vehicle (control) or p300 inhibitor (20 μM) were differentiated for 3 days. The mRNA levels of the indicated genes were determined using qRT-PCR. Data are represented as mean ± SD (n = 3). (**F**) The expression levels of MyHC I, MyHC II, and Mef2c in a vehicle (control)-treated or p300 inhibitor (20 μM)-treated C2C12 cells were measured using western blotting after differentiation in the presence of 2% horse serum for 3 days. Actin was used as a loading control. (**H**) C2C12 cells treated with vehicle (control) or rC1qbp (500 nM) were differentiated in the presence of 2% horse serum for 5 days. ChIP assays were performed as described in (**B**). Data are represented as mean ± SD (n = 3). (**I**) C2C12 cells treated with vehicle (control) or 500 nM rC1qbp were differentiated for 5 days. The total lysates (upper), cytosol (lower left), and nuclear (lower right) fractions were prepared and analyzed by western blotting. Actin, tubulin, and lamin A/C were used as loading controls. Original blots in (**F**,**I**) are presented in Supplementary Fig. S6. *P < 0.05; **P < 0.01; ***P < 0.001.

A precise molecular mechanism of NFATc1 in muscle myogenic differentiation has not been previously reported. As NFATc1 directly binds to the histone acetyltransferase (HAT) p300^19^, the occupancy of NFATc1 and p300 at the promoter regions of the NFATc1 target genes was examined using chromatin immunoprecipitation (ChIP) assays (Fig. 6A and Supplementary Fig. S1A,B). NFATc1 knockdown suppressed p300 occupancy at the promoter of the target genes (Fig. 6B and Supplementary Fig. S1C,F). Consistently, treatment with a p300 inhibitor suppressed NFATc1 occupancy (Fig. 6C and Supplementary Fig. S1D,G). Moreover, NFATc1 knockdown and treatment with a p300 inhibitor significantly downregulated histone H3 acetylation but not the levels of H3 at the NFATc1 target gene promoters (Fig. 6B,C, and Supplementary Fig. S1C,D,F,G). These findings suggest that histone H3 acetylation via p300 may be important for the transcriptional activity of NFATc1 at its target genes. Next, the effect of p300 on in vitro myogenesis was examined. Treatment with a p300 inhibitor significantly suppressed myogenic differentiation of C2C12 cells (Fig. 6D). Additionally, the p300 inhibitor downregulated the expression levels of myogenic differentiation markers (Fig. 6E,F) and NFATc1 target genes (Fig. 6G). As expected, rC1qbp treatment suppressed NFATc1 and p300 occupancies and histone H3 acetylation level at the promoter of the target genes (Fig. 6H and Supplementary Fig. S1E,H). Finally, western blot revealed that rC1qbp suppressed p300 expression in C2C12 cells, especially in their nuclear fractions (Fig. 6I), suggesting that rC1qbp may inhibit myogenesis via suppression of p300 expression and/or its nuclear translocation. Regarding the mechanism of action of C1qbp, these findings indicate that the NFATc1/p300 complex regulates the expression of NFATc1 target genes and myogenic differentiation.

**Figure 7:**
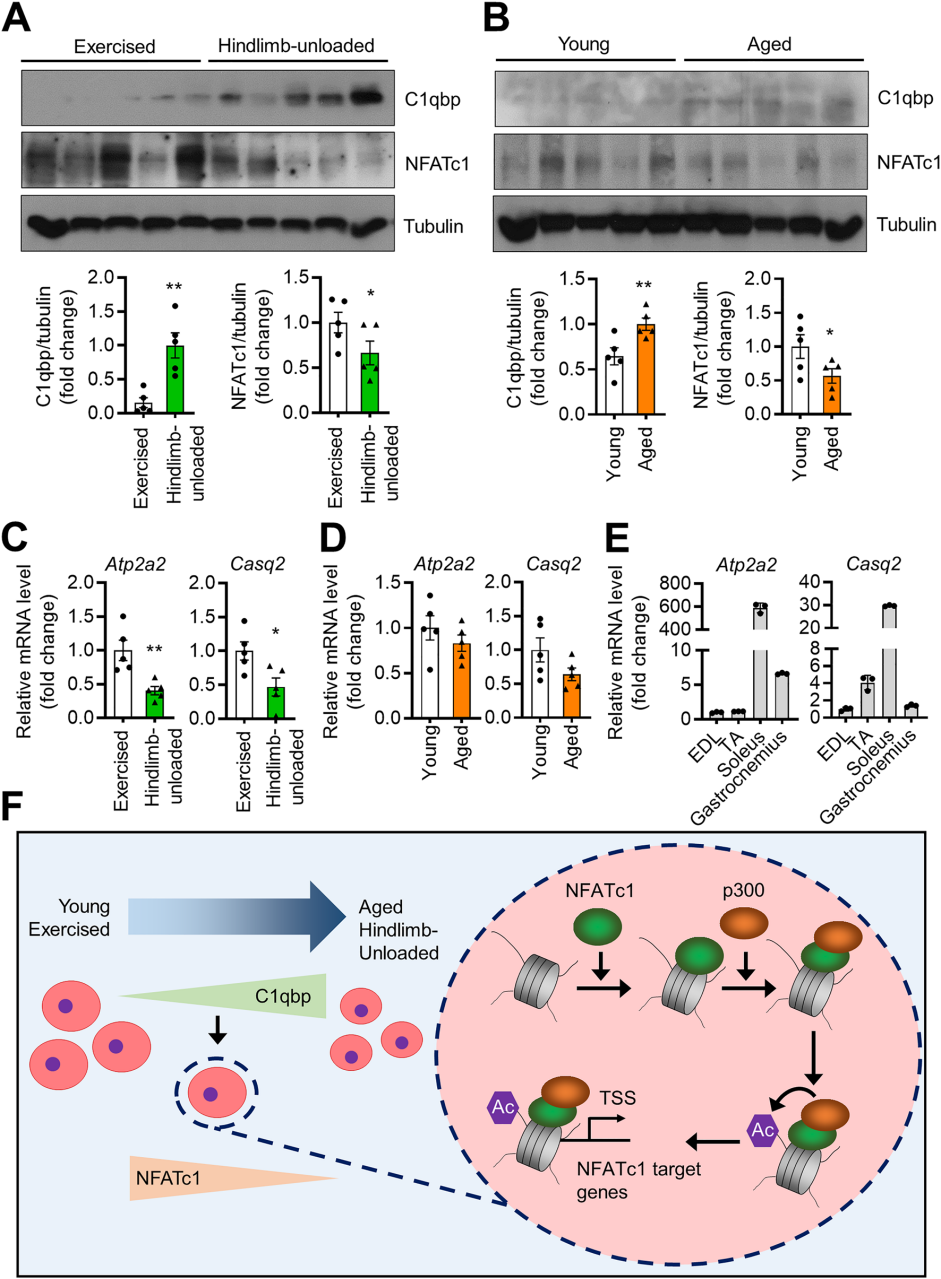
Expression levels of NFATc1 and C1qbp in mice with muscle loss. (**A**) Gastrocnemius muscles from exercised and hindlimb-unloaded male C57BL/6 mice were prepared. The expression levels of NFATc1 and C1qbp were analyzed using western blotting (upper). The protein levels were quantified and normalized to tubulin levels (lower). Data are represented as mean ± SEM (n = 5 mice). (**B**) Gastrocnemius muscles of 7-month-old (young) and 19-month-old (aged) male C57BL/6 mice were subjected to western blotting analysis using anti-NFATc1 and anti-C1qbp antibodies. The quantification of the protein levels is shown in the lower panels. Tubulin was used as a loading control. Data are represented as mean ± SEM (n = 5 mice). (**C**,**D**) Gastrocnemius muscles were extracted from exercised/hindlimb-unloaded (**C**) and young/aged (**D**) male C57BL/6 mice. The mRNA expression levels of *Atp2a2* and *Casq2* were measured using qRT-PCR. Data are represented as mean ± SEM (n = 5 mice). (**E**) The mRNA expression levels of *Atp2a2* and *Casq2* in mouse muscle tissues were measured using qRT-PCR. Data are represented as mean ± SD (n = 3). (**F**) A proposed model for the suppression of myogenic differentiation by C1qbp. Original blots in (**A**,**B**) are presented in Supplementary Fig. S7. *P < 0.05; **P < 0.01.

We expected an inverse expressional correlation between C1qbp and NFATc1 as C1qbp treatment suppressed NFATc1 expression. To demonstrate this in vivo, the skeletal muscle expression levels of C1qbp and NFATc1 in hindlimb-unloaded and aged mice were compared with those in exercised and young mice, respectively. Consistent with the results of the proteomics analysis, the expression levels of C1qbp in the gastrocnemius of hindlimb-unloaded and aged mice were significantly higher than those of exercised and young mice, respectively (Fig. 7A,B). Conversely, the expression levels of NFATc1 in the gastrocnemius of hindlimb-unloaded and aged mice were significantly lower than those of exercised and young mice, respectively. However, this finding was not observed in the tibialis anterior, suggesting that it may vary by muscle types (Supplementary Fig. S2A,B). Additionally, the expression levels of NFATc1 target genes were determined. The muscle levels of *Atp2a2* and *Casq2* in hindlimb-unloaded mice were lower than those in exercised mice (Fig. 7C), although differences in the muscle levels of *Atp2a2* and *Casq2* between young and aged mice were not statistically significant (Fig. 7D). NFATc1 is the main transcription regulator in type 1 myofibers^20–22^. Consistently, the expression levels of *Atp2a2* and *Casq2* were the highest in the soleus (Fig. 7E).

## Discussion

This study aimed to identify a novel factor involved in skeletal muscle physiology using an untargeted proteomic approach to analyze the muscles of mice exhibiting muscle atrophy. The skeletal muscle expression level of C1qbp in hindlimb-unloaded and aged mice was higher than that in exercised and young mice, respectively. Based on the results of in vitro and in vivo experiments, we propose a model in which extracellular C1qbp functions as a negative regulator of myogenic differentiation (Fig. 7F). Unloading and aging increase the expression of C1qBP expression in muscles, which causes muscle atrophy. C1qbp inhibits myogenic differentiation by mechanisms both through attenuation of NFATc1 expression and independently of NFATc1. NFATc1 binds to the promoter of its target genes and recruits the HAT p300 through interaction. The p300-mediated upregulation of histone H3 acetylation promotes the transactivation of NFATc1 at its target genes. To the best of our knowledge, this is the first study to report the role of extracellular C1qbp in skeletal muscle physiology.

This study demonstrated that the expression of C1qbp was highly detected in the muscle tissues, which was consistent with the results of a previous study^23^. C1qbp is mainly expressed in the mitochondrial matrix^24^, although its expression has been reported in other subcellular compartments, such as the nucleus, endoplasmic reticulum, golgi, and cell membrane^25–27^. C1qbp can also be secreted into the extracellular matrix^25–27^. The levels of C1qbp in CM from muscle cells, especially during early differentiation, were examined. Secreted C1qbp is reported to regulate various functions, including angiogenesis, vascular permeability, and inflammation^28,29^. This study demonstrated that C1qbp acts directly on muscle cells to suppress their differentiation. Furthermore, secreted C1qbp from activated endothelial cells can induce bradykinin receptor 1 expression and consequently upregulate proinflammatory cytokines, such as TNFα, IL-1β, and IL-6, and the chemokines IL8 and MCP1^13,30,31^. Cytokines, including IL6 and TNFα, promote muscle atrophy^32,33^. Thus, secreted C1qbp may be directly involved in the process of muscle atrophy by promoting the release of cytokines, which must be verified in future studies.

C1qbp was upregulated in the skeletal muscles of aged and hindlimb-unloaded mice. Treatment with rC1qbp decreased myogenic muscle mass. This suggests that muscle atrophy caused by aging and lack of exercise may be due to the upregulation of C1qbp. Therefore, anti-C1qbp treatment modalities, such as anti-C1qbp blocking antibodies and C1qbp inhibitors, may be developed to overcome diseases associated with muscle atrophy. Sarcopenia is a major public health issue. However, anti-sarcopenic drugs have not been approved. The results of this study revealed a novel therapeutic target for diseases associated with muscle atrophy, including sarcopenia.

Previous studies have revealed that the calcineurin/NFAT pathway plays an important role in regulating skeletal myoblast differentiation^15,20–22^. The expression of NFATc1 is the highest in adult skeletal muscle and myotubes^21^. NFATc1 plays an important regulatory role in fiber type switching with exercise stimulation, as well as in regulating the expression of slow fiber-related genes^22^. Consistently, NFATc1 expression in the exercised mice was upregulated when compared with that in the hindlimb-unloaded mice. Consequently, the expression levels of *Atp2a2* and *Casq2*, which are slow type I fiber-related genes, were upregulated in the exercised muscles. These findings indicate that NFATc1 expression is upregulated in response to exercise stimuli to promote slow fiber formation by upregulating the related gene expression. However, the expression levels of *Atp2a2* and *Casq2* did not change with aging as shown in Fig. 7D although NFATc1 expression was downregulated in the aged muscle. This suggests that the roles of C1qbp and NFATc1 in aging-induced fiber type switch may be independent of *Atp2a2* and *Casq2* expression. Thus, the mechanism underlying aging-related changes in slow fiber-related gene expression must be examined.

This study demonstrated that NFATc1 enhances the expression of slow fiber-related genes, such as *Atp2a2* and *Casq2* by recruiting the transcription coactivator p300. Furthermore, p300 promoted the transactivation of slow fiber-related genes by acetylating histone H3 at the promoter of its target genes and upregulated myogenic differentiation. This indicates that p300 is required for myogenic differentiation and the formation of slow-type fibers. These findings are consistent with those of previous studies, which reported that p300-mediated histone H3 acetylation plays a critical role in regulating myoblast differentiation^34^ and that p300 enhanced slow myosin heavy chain I/b gene expression by binding to its promoter and upregulated NFTAc1 transcriptional activity^19^.

Since C1qbp suppressed NFATc1 expression, we assumed that the inhibition of myogenic differentiation by C1qbp was due to inhibition of NFATc1 expression. However, NFATc1 overexpression only partially, but not completely, restored the myogenic differentiation suppressed by rC1qbp. This suggests that the inhibitory effect of C1qbp may be caused by two distinct mechanisms, such as inhibition of NFATc1 expression and an NFATc1-independent mechanism. One factor other than NFATc1 could be p300. Because C1qbp suppressed p300 levels independently of NFATc1, it is possible that C1qbp inhibited myogenic differentiation even when NFATc1 was abundant. In addition, we cannot exclude the possibility that NFATc1 overexpression may not be sufficient to eliminate all effects of C1qbp on myogenic differentiation.

Ma et al.^35^. recently reported that C1qbp silencing reduced myoblast differentiation and proliferation and induced cell apoptosis in sheep myoblasts. Moreover, the expression of C1qbp in myotubes was higher than that in myoblasts during their differentiation^35^. However, this study demonstrated contrasting results for the expression pattern of C1qbp during myogenic differentiation. C1qbp overexpression rather partially inhibited myogenesis in murine myoblasts. The exact cause of these inconsistent results between this study and Ma et al. is unclear. The different tested cells and tissues used between the two studies may have contributed to inconsistent results.

Krt14 was observed to be expressed in skeletal muscle by proteomics screen, but not by immunohistochemical staining and qRT-PCR. It is detectable in very small amounts in proteomic analysis, but may not be detectable in immunohistochemical analysis due to antibody specificity and detection limits. However, it was also not detected by qRT-PCR, suggesting that it is more likely that keratin 14, which is abundant in the skin^36^, may be contaminated during the harvesting of muscle tissue. In any case, these findings suggest that it is essential to double-check factors identified by proteomic analysis with traditional methods such as immunohistochemistry and qRT-PCR.

We observed that C1qbp overexpression also caused more expression in CM. However, C1qbp overexpression only partially inhibited myogenic differentiation in the present study. It seems likely that the amount of C1qbp secreted extracellularly may not be sufficient to strongly inhibit myogenic differentiation. In addition, we observed that C1qbp treatment at concentrations of 5 to 500 nM suppressed myogensis in vitro. To our knowledge, there is no study about the circulating level of C1qbp, and therefore it is not known this amount of C1qbp is physiological. Further studies are needed to determine what the concentration of C1qbp in the blood is and whether sarcopenia correlates with the level. Finally, as shown in Fig. 7, the in vivo data showed opposite expression patterns of C1qbp and NFATc1 in the gastrocnemius muscles. However, this does not really prove a causal relationship between C1qbp and NFATc1 in relation to muscle differentiation. Further studies using rC1qbp intervention or genetically-engineered mice are needed to prove causality.

In conclusion, this study demonstrated a novel role of extracellular C1qbp, which involves the suppression of myogenesis by inhibiting the NFATc1/p300 complex. Thus, anti-C1qbp treatment modalities can be developed as a novel therapeutic strategy for muscle loss.

## Methods

### Animals

All experimental animal protocols were approved by the Institutional Animal Care and Use Committee of the Asan Institute for Life Sciences (No. 2016-12-035). All experiments followed the ARRIVE guidelines 2.0. Mice were allowed to acclimatize to specific pathogen-free conditions at the Asan Institute for Life Sciences and provided access to rodent chow and water ad libitum. Euthanasia procedure was performed in accordance with the guidelines of the Institutional Animal Care and Use Committee of the Asan Institute for Life Sciences. Before euthanasia, all mice were anesthetized with Zoletil (50 mg/kg, i.p.) and Xylazine (10 mg/kg, i.p.), and their gastrocnemius muscles were obtained.

#### Exercised mice versus hindlimb-unloaded mice

Male C57BL/6 mice (n = 5) aged 7 weeks (Orient Bio Inc., Seongnam, Republic of Korea) were subjected to exercise with a treadmill running at a speed of 15 m/min at a 10° incline for 30 min/day for 3 weeks^37,38^. In the hindlimb-unloaded group, male C57BL/6 mice (n = 5) aged 7 weeks underwent tail suspension for hindlimb unloading for 3 weeks. To maximize the effects of exercise restriction, mice running on a treadmill, as supernormal controls, were compared with the hindlimb-unloaded mice^39,40^. Mice in both groups were euthanized at 10 weeks of age, and their gastrocnemius muscle were obtained.

#### Young mice versus aged mice

Male C57BL/6 mice (n = 7) aged 19 months were purchased from the Korea Research Institute of Bioscience and Biotechnology (Daejeon, Republic of Korea). Meanwhile, male C57BL/6 mice (n = 8) aged 7 months served as young controls.

### Proteomic analyses of gastrocnemius muscles

Mouse muscle tissue samples in 5% sodium dodecyl sulfate buffer were lysed using an S220 Focused-ultrasonicator (Covaris, USA), following the manufacturer’s instructions. The lysates were subjected to S-trap-based tryptic digestion with a trypsin/LysC:protein mixture (in the ratio 1:25) for 12 h at 37 °C (Promega, Madison, WI, USA). The dried peptides were reconstituted in 0.1% formic acid, and the peptide concentration was determined by measuring the absorbance at 205 nm wavelength using a NanoDrop One spectrophotometer (Thermo Fisher Scientific, Wilmington, DE, USA). The peptide (40 μg) of each sample was dried and stored at − 80 °C until further use. The digested peptide was measured twice using the Dionex UltiMate 3000 RSLCnano system coupled with a Q Exactive mass spectrometer (Thermo Fisher Scientific). The liquid chromatography gradient and data-dependent acquisition-mass spectrometry parameters followed a previously published method^41,42^. Additionally, the acquired mass spectrometry spectra were searched using Sequest HT on Proteome discoverer (version 2.3; Thermo Fisher Scientific) against the SwissProt mouse proteome sequence database (May 2020). The search parameters were set as default including cysteine carbamidomethylation as a fixed modification, and N-terminal acetylation and methionine oxidation as variable modifications with two miscleavages. Peptides were identified based on a search with an initial mass deviation of the precursor ion of up to 10 ppm with the allowed fragment mass deviation set to 20 ppm. When assigning proteins to peptides, both unique and razor peptides were used. Label-free quantitation was performed using peak intensity for unique peptides of each protein. The mass spectrometry proteomics data have been deposited to the ProteomeXchange Consortium via the PRIDE^43^ partner repository with the dataset identifier PXD043715.

### Constructs and antibodies

For mammalian expression, the cDNA encoding of C1qbp was PCR-amplified and ligated into the correct reading frames of pIRES-neo (Clontech Laboratories, Inc., Mountain View, CA, USA) containing 5′-FLAG and hemagglutinin (HA) coding sequences. The mouse Pfn2 (MG51608-NY) mammalian expression construct was obtained from Sino biological Inc. (Beijing, China). The following antibodies were used in this study: anti-C1qbp (ab24733) and Fast-MyHC (ab91506) antibodies (Abcam, Cambridge, UK); anti-Bax (orb213598), and anti-Krt14 antibodies (orb214169) (Biorbyt, Cambridge, UK); anti-MyCH I (M8421), anti-MyCH II (M1570), anti-Actin (A3854), and anti-tubulin antibodies (T9026) (Sigma, St. Louis, MO, USA); anti-Mef2c (#5030) and anti-histone H3 (#4620) antibodies (Cell Signaling Technology, MA, USA); anti-Lamin A/C (39287) antibody (Thermo Fisher Scientific); anti-TRAP (sc-28204) and anti-NFATc1 antibodies (sc-7294) (Santacruz Biotechnology, Dallas, TX, USA); anti-NFATc3 (GTX133744) (GeneTex, Irvine, CA, USA); anti-p300 (RW105) (Novus Biologicals, USA), and anti-acetyl-histone H3 antibodies (06-599) (Merck, Darmstadt, Germany).

### Cell culture and myoblast differentiation

C2C12 cells were cultured in Dulbecco’s modified Eagle’s medium (DMEM) supplemented with 10% fetal bovine serum (Gibco, Grand Island, NY, USA). For the myoblast differentiation assay, the cells (3 × 10^5^ cells) were seeded onto a 35-mm culture dish and incubated for 1 day. When the culture reached 90%–100% confluence, the cells were replenished with DMEM containing 2% horse serum every 24 h. The myotube areas, nuclei number per myotube, and fusion index were analyzed using the Image-Pro Plus (version 6.0; Media Cybernetics, Rockville, MD, USA) and Image J (version 1.54 g; National Institutes of Health, Bethesda, MD, USA) programs.

### siRNA experiments, inhibition, and cell viability

For silencing NFATc1 and NFATc3, DNA oligonucleotides encoding shRNAs specific for NFATc1 (5′-CCCGTCCAAGTCAGTTTCTAT-3′) and NFATc3 mRNA coding regions (5′-GCTCACATTGTCCTTGAAGTT-3′) were annealed and ligated into the lentiviral expression vector pLKO.1 (Addgene). Lentivirus particles were generated in HEK-293T cells by co-transfecting plasmids encoding VSV-G, NL-BH, and the shRNAs using Lipofectamine 2000 reagent (Invitrogen, Carlsbad, CA, USA), following the manufacturer’s instructions. C2C12 cells were infected by these viruses and selected with 2 µg/ml puromycin (Sigma). For inhibition of p300, cells were cultured with dimethyl sulfoxide or 20 μM MMP9 inhibitor C646 (Apexbio, Houston, TX, USA) and subjected to myogenic differentiation. Cell viability was investigated using a commercially available cell counting kit-8 (CCK-8; Dojindo, Kumamoto, Japan), following the manufacturer’s instructions.

### Hematoxylin and eosin staining and immunofluorescence

Muscle tissues were dissected, mounted, frozen, and sectioned to a thickness of 10 μm using the CM1860 cryostat (Leica Microsystems, Wetzlar, Germany). The sections were stained with hematoxylin and eosin. For immunofluorescence staining, the frozen sections were rehydrated, boiled with 10 mM sodium citrate buffer for 10 min, and blocked with 2% bovine serum albumin (BSA) for 1 h. Next, the sections were incubated with anti-C1qbp (1:50) antibodies overnight at 4 °C, followed by incubation with Alexa Fluor 555-conjugated secondary antibodies (Invitrogen, 1:400). Nuclei were counterstained with 4′,6-diamidino-2-phenylindole (DAPI) (Life Technologies, Carlsbad, CA, USA).

### Transfection of constructs

C2C12 cells (2.5 × 10^4^ cells) were seeded on a 4-well slide chamber (Thermo Fisher Scientific) for 24 h and then transfected with vector only (control), Pfn2 (positive control) and C1qbp constructs for 4 h using a Lipofectamine 3000 transfection reagent (Invitrogen), following the manufacturer’s instructions. After washing, the cells were incubated DMEM with 10% FBS for 24 h. The cells were differentiated with DMEM containing 2% horse serum for 3 and 4 days for MyHC immunostaining.

### Treatment of blocking antibody

C2C12 cells (2.5 × 10^4^ cells) were seeded on a 4-well slide chamber (Thermo Fisher Scientific) and further cultured for 2 days. The cells were treated with normal mouse IgG (10 μg/ml), C1qbp-blocking antibody (10 μg/ml, Abcam ab24733), IgG plus rC1qbp (500 nM) and C1qbp-blocking antibody plus rC1qbp in DMEM containing 2% horse serum every day. Four days later, cells were stained for MyHC immunostaining.

### Infection of retrovirus

Retrovirus-packaging Plat-E cells were transfected with constitutively active form of NFATc1 (ca-NFATc1) constructs^44^. After 2 days, retroviral supernatant was collected from culture media. C2C12 cells (2.5 × 10^4^ cells) were seeded on a 4-well slide chamber for 24 h and cultured with retroviral supernatant and polybrene (10 μg/ml) for 8 h. The cells were removed the retroviral supernatant and incubated with the culture medium for 24 h. The cells were treated with rC1qbp (500 nM) in DMEM containing 2% horse serum every day. Four days later, cells were stained for MyHC immunostaining.

### Fraction of cytosol and nucleus

C2C12 cells (1 × 10^5^ cells) were seeded on a 35-mm culture plate for 2 days. The cells were treated with rC1qbp (500 nM) and then differentiated into myotubes in DMEM with 2% horse serum for 5 days. The cells were subjected to prepare nuclear and cytosolic fractions using NE-PER nuclear cytoplasmic extraction reagents (Thermo Fisher Scientific 78833) according to the manufacturer’s instructions. The isolated cytosolic and nuclear fractions were analyzed by western blot using a NFATc1 antibody.

### Immunohistochemistry and immunostaining

The paraffin-embedded sections were subjected to immunohistochemical analysis, following standard protocols. The sections were blocked and incubated with anti-C1qbp (1:50), anti-Bax (1:50), anti-Krt14 (1:50), anti-Fast MyHC (1:50), and anti-TRAP (1:50) antibodies overnight at 4 °C, followed by incubation with the Dako Envision + System-horseradish peroxidase-labeled polymer secondary antibody system (Dako, Carpinteria, CA, USA), following the manufacturer’s instructions. All sections were counterstained with hematoxylin. For MyHC immunostaining, cells were fixed with 4% paraformaldehyde for 15 min and permeabilized with 0.5% Triton X-100 in phosphate-buffered saline (PBS) for 10 min. Cells were blocked with 2% BSA in PBS and incubated with anti-MyHC antibodies (1:1000) overnight at 4 °C. Next, the cells were incubated with anti-mouse Alexa Fluor 555 antibodies (1:1000) and counterstained with DAPI. Fluorescent images were captured using a Zeiss LSM 710 confocal microscope (Carl Zeiss, Germany).

### Western blotting

Cells were washed with PBS and lysed in radioimmunoprecipitation assay buffer containing a protease inhibitor mixture (Sigma). The proteins were subjected to sodium dodecyl sulfate–polyacrylamide gel electrophoresis. The resolved proteins were transferred onto a 0.2 μm nitrocellulose membrane and probed with primary antibodies. Next, the membrane was probed with appropriate secondary antibodies (Cell Signaling Technology). Immunoreactive signals were developed with enhanced chemiluminescence reagents (PerkinElmer, Waltham, MA, USA). Band density was determined using an Image Guage (version 4.0; Fujifilm, Tokyo, Japan). Some membranes were cut and then subjected to western blot analysis.

### qRT-PCR analysis

Total RNA was isolated using TRIzol reagent (Invitrogen), following the manufacturer’s instructions. The isolated RNA was then reverse-transcribed into complementary DNA (cDNA) using the cDNA synthesis kit (Invitrogen). The cDNA was subjected to qRT-PCR analysis using the SYBR Green PCR kit (Roche Diagnostics, Manheim, Germany), following the manufacturer’s instructions. The primers used for qRT-PCR analysis are listed in Supplementary Table S3.

### ChIP analysis

ChIP analysis was performed using the ChIP assay kit (Millipore) as described previously^45^. Immunoprecipitated DNA was quantified using qRT-PCR with the promoter regions of the *Atp2a2*, *Casq2*, and *Stab2*. The primers for qRT-PCR analysis are listed in Supplementary Table S4.

### Statistical analysis

All quantitative data are presented as mean ± standard deviation (SD) or standard error of mean (SEM) as indicated. Data were analyzed using Student’s two-tailed *t*-test or one/two-way analysis of variance, followed by Bonferroni’s comparison test. GraphPad Prism (version 8.0; GraphPad Software Inc., San Diego, CA, USA) was used for all statistical analyses. Differences were considered significant at *P* < 0.05.

### Supplementary Information

Supplementary Information.

## Data Availability

Data are available via ProteomeXchange with identifier PXD043715. All data generated or analyzed during this study are included in this published article and its supplementary information files.
