# Supplementary material for: Extracellular C1qbp inhibits myogenesis by suppressing NFATc1
Source: Sci Rep. 2024 Jul 8;14:15678. doi: 10.1038/s41598-024-66549-1 (PMC11231330; doi:10.1038/s41598-024-66549-1)
Supplement: Supplementary file 1 — Supplementary Information. [file 41598_2024_66549_MOESM1_ESM.pdf]

Supplemental Information

Extracellular C1qbp inhibits myogenesis by suppressing NFATc1

Jin-Man Kim, Ho Kyoung Kim, Han Jin Cho, Sung-Ah Moon, Yewon Kim, Jeong Yeon Hong, Seung Hun Lee, Kyunggon Kim, and Jung-Min Koh

Supplementary Table S1. The list of up-regulated proteins in muscles of hindlimb-unloaded mice vs. exercised mice

Acession	Description	Gene
P63028	Translationally-controlled tumor protein	Tpt1
Q9Z172	Small ubiquitin-related modifier 3	Sumo3
Q07813	Apoptosis regulator BAX	Bax
E9Q557	Desmoplakin	Dsp
P02533	Keratin, type I cytoskeletal 14	Krt14
Q9CR61	NADH dehydrogenase [ubiquinone] 1 beta subcomplex subunit 7	Ndufb7
Q9QZ47	Troponin T3, fast skeletal muscle	Tnnt3
P02535	Keratin, type I cytoskeletal 10	Krt10
P21126	Ubiquitin-like protein 4A	Ubl4a
Q9WV55	Vesicle-associated membrane protein-associated protein A	Vapa
Q9DBJ1	Phosphoglycerate mutase 1	Pgam1
Q9CQM9	Glutaredoxin-3	Glrx3
Q8K3J1	NADH dehydrogenase [ubiquinone] iron-sulfur protein 8, mitochondrial	Ndufs8
P05413	Fatty acid-binding protein 3, heart	Fabp3
P14152	Malate dehydrogenase 1, cytoplasmic	Mdh1
Q9D6J6	NADH dehydrogenase [ubiquinone] flavoprotein 2, mitochondrial	Ndufv2
Q9WUB3	Glycogen phosphorylase, muscle form	Pygm
Q62446	Peptidyl-prolyl cis-trans isomerase 3	Fkbp3
O35658	Complement component 1 Q subcomponent-binding protein, mitochondrial	C1qbp
P13541	Myosin-3	Myh3
P02538	Keratin, type II cytoskeletal 6A	Krt6a
Q06185	ATP synthase subunit e, mitochondrial	Atp5me
Q00898	Alpha-1-antitrypsin 1-5	Serpina1e
P09411	Phosphoglycerate kinase 1	Pgk1
Q61091	Frizzled-8	Fzd8
P70296	Phosphatidylethanolamine-binding protein 1	Pebp1
P08779	Keratin, type I cytoskeletal 16	Krt16
Q9QWL7	Keratin, type I cytoskeletal 17	Krt17
Q9JKS4	LIM domain-binding protein 3	Ldb3
Q8BSY0-2	Isoform 2 of Aspartyl/asparaginyl beta-hydroxylase	Asph
Q99LM3	Smoothelin-like protein 1	Smtnl1
Q9WTQ5	A-kinase anchor protein 12	Akap12
P11087	Collagen alpha-1(I) chain	Col1a1
P01592	Immunoglobulin J chain	Jchain
Q8CGP2-2	Isoform 2 of Histone H2B type 1-P	H2bc22
P04370	Myelin basic protein	Mbp
Q9D6R2	Isocitrate dehydrogenase [NAD] 3 subunit alpha, mitochondrial	Idh3a
Q3MI48	Junctional sarcoplasmic reticulum protein 1	Jsrp1
P00493	Hypoxanthine-guanine phosphoribosyltransferase1	Hprt1
P68372	Tubulin beta-4B chain	Tubb4b
P97371	Proteasome activator complex subunit 1	Psme1
P13647	Keratin, type II cytoskeletal 5	Krt5
P14602	Heat shock protein beta-1	Hspb1
P02088	Hemoglobin subunit beta-1	Hbb-b1
O08715-4	Isoform 4 of A-kinase anchor protein 1, mitochondrial	Akap1
Q9R0Y5-2	Isoform 2 of Adenylate kinase isoenzyme 1	Ak1
P19536	Cytochrome c oxidase subunit 5B, mitochondrial	Cox5b
P05977	Myosin light chain 1/3, skeletal muscle isoform	Myl1
Q9CQA3	Succinate dehydrogenase [ubiquinone] iron-sulfur subunit, mitochondrial	Sdhb
P97457	Myosin regulatory light chain 2, skeletal muscle isoform	Mylpf
Q9D1X0	Nucleolar protein 3	Nol3
O54931	A-kinase anchor protein 2	Akap2
P09528	Ferritin heavy chain 1	Fth1

Acession	Description	Gene
P19783	Cytochrome c oxidase subunit 4 isoform 1, mitochondrial	Cox4i1
Q01149	Collagen alpha-2(I) chain	Col1a2
P35908	Keratin, type II cytoskeletal 2 epidermal	Krt2
O08539	Myc box-dependent-interacting protein 1	Bin1
Q8CJ53	Cdc42-interacting protein 4	Trip10
Q9CZ13	Cytochrome b-c1 complex subunit 1, mitochondrial	Uqcrc1
Q9CRB6	Tubulin polymerization-promoting protein family member 3	Tppp3
Q9D882	Uncharacterized protein FAM241B	Fam241b
P13542	Myosin-8	Myh8
Q8R429	Sarcoplasmic/endoplasmic reticulum calcium ATPase 1	Atp2a1
Q922J3	CAP-Gly domain-containing linker protein 1	Clip1
O70435	Proteasome subunit alpha type-3	Psma3
P08122	Collagen alpha-2(IV) chain	Col4a2
P17751	Triosephosphate isomerase 1	Tpi1
Q5DTJ9	Myopalladin	Mypn
Q9DCW4	Electron transfer flavoprotein subunit beta	Etfb
P99027	60S acidic ribosomal protein P2	Rplp2
Q70KF4	Cardiomyopathy-associated protein 5	Cmya5
Q9D6J5	NADH dehydrogenase [ubiquinone] 1 beta subcomplex subunit 8, mitochondrial	Ndufb8
P04264	Keratin, type II cytoskeletal 1	Krt1
P20801	Troponin C2, skeletal muscle	Tnnc2
O70624	Myocilin	Myoc
P07310	Creatine kinase M-type	Ckm
Q3TJD7-2	Isoform 2 of PDZ and LIM domain protein 7	Pdlim7
P12787	Cytochrome c oxidase subunit 5A, mitochondrial	Cox5a
P16015	Carbonic anhydrase 3	Ca3
Q9Z2U0	Proteasome subunit alpha type-7	Psma7
Q9CQ75	NADH dehydrogenase [ubiquinone] 1 alpha subcomplex subunit 2	Ndufa2
P28740-1	Isoform 1 of Kinesin-like protein KIF2A	Kif2a

Supplementary Table S2. The list of up-regulated proteins in muscles of aged mice vs. young mice

Acession	Description	Gene
P63028	Translationally-controlled tumor protein	Tpt1
Q9D882	Uncharacterized protein FAM241B	Fam241b
Q8CGP2-2	Isoform 2 of Histone H2B type 1-P	H2bc22
Q9DCX2	ATP synthase subunit d, mitochondrial	Atp5h
Q8K3J1	NADH dehydrogenase [ubiquinone] iron-sulfur protein 8, mitochondrial	Ndufs8
O08807	Peroxiredoxin-4	Prdx4
Q9CQM5	Thioredoxin domain-containing protein 17	Txndc17
P11352	Glutathione peroxidase 1	Gpx1
O70548	Telethonin	Tcap
P19536	Cytochrome c oxidase subunit 5B, mitochondrial	Cox5b
P17751	Triosephosphate isomerase 1	Tpi1
P97315	Cysteine and glycine-rich protein 1	Csrp1
Q9CQ75	NADH dehydrogenase [ubiquinone] 1 alpha subcomplex subunit 2	Ndufa2
O70624	Myocilin	Myoc
Q6ZQ73	Cullin-associated NEDD8-dissociated protein 2	Cand2
P08226	Apolipoprotein E	Apoe
P09541	Myosin light chain 4	Myl4
Q9JI75	Ribosyldihydronicotinamide dehydrogenase [quinone]	Nqo2
P56391	Cytochrome c oxidase subunit 6B1	Cox6b1
Q9WUB3	Glycogen phosphorylase, muscle form	Pygm
Q9CQA3	Succinate dehydrogenase [ubiquinone] iron-sulfur subunit, mitochondrial	Sdhb
Q8BSY0-2	Isoform 2 of Aspartyl/asparaginyl beta-hydroxylase	Asph
P02088	Hemoglobin subunit beta-1	Hbb-b1
P05413	Fatty acid-binding protein 3, heart	Fabp3
P19783	Cytochrome c oxidase subunit 4 isoform 1, mitochondrial	Cox4i1
Q9CPU0	Lactoylglutathione lyase 1	Glo1
P04247	Myoglobin	Mb
P56213	FAD-linked sulfhydryl oxidase ALR	Gfer
Q9D6R2	Isocitrate dehydrogenase [NAD] 3 subunit alpha, mitochondrial	Idh3a
Q61554	Fibrillin-1	Fbn1
Q07813	Apoptosis regulator BAX	Bax
Q91Z83	Myosin-7	Myh7
Q3TJD7-2	Isoform 2 of PDZ and LIM domain protein 7	Pdlim7
Q9CR61	NADH dehydrogenase [ubiquinone] 1 beta subcomplex subunit 7	Ndufb7
P20029	78 kDa glucose-regulated protein	Hspa5
P13412	Troponin I2, fast skeletal muscle	Tnni2
P13542	Myosin-8	Myh8
Q9Z2U1	Proteasome subunit alpha type-5	Psma5
P0C6F1	Dynein heavy chain 2, axonemal	Dnah2
P99027	60S acidic ribosomal protein P2	Rplp2
Q9Z2S7-4	Isoform 4 of TSC22 domain family protein 3	Tsc22d3
O08715-4	Isoform 4 of A-kinase anchor protein 1, mitochondrial	Akap1
P09542	Myosin light chain 3	Myl3
Q9Z1P6	NADH dehydrogenase [ubiquinone] 1 alpha subcomplex subunit 7	Ndufa7
Q9D6J6	NADH dehydrogenase [ubiquinone] flavoprotein 2, mitochondrial	Ndufv2
P05977	Myosin light chain 1/3, skeletal muscle isoform	Myl1
P02533	Keratin, type I cytoskeletal 14	Krt14
Q62446	Peptidyl-prolyl cis-trans isomerase FKBP3	Fkbp3
P27661	Histone H2AX	H2afx
Q00623	Apolipoprotein A-I	Apoa1
P01942	Hemoglobin subunit alpha	Hba
Q64191	N(4)-(beta-N-acetylglucosaminyl)-L-asparaginase	Aga
P32848	Parvalbumin alpha	Pvalb

Acession	Description	Gene
Q06185	ATP synthase subunit e, mitochondrial	Atp5me
Q3UHX2	28 kDa heat- and acid-stable phosphoprotein	Pdap1
Q9QXS1	Plectin	Plec
P47857-3	Isoform 3 of ATP-dependent 6-phosphofructokinase, muscle type	Pfkm
Q6ZWQ0	Nesprin-2	Syne2
Q9CQ60	6-phosphogluconolactonase	Pgls
Q8VCF0	Mitochondrial antiviral-signaling protein	Mavs
P46471	26S proteasome regulatory subunit 7	Psmc2
O70622-2	Isoform 2 of Reticulon-2	Rtn2
Q9QXT0	Protein canopy homolog 2	Cnpy2
Q9CRB6	Tubulin polymerization-promoting protein family member 3	Tppp3
Q8C7E7	Starch-binding domain-containing protein 1	Stbd1
O09061	Proteasome subunit beta type-1	Psmb1
Q9Z2U0	Proteasome subunit alpha type-7	Psma7
O35114	Lysosome membrane protein 2	Scarb2
P06728	Apolipoprotein A-IV	Apoa4
P16858	Glyceraldehyde-3-phosphate dehydrogenase	Gapdh
Q9JKK8	Serine/threonine-protein kinase ATR	Atr
P47968	Ribose-5-phosphate isomerase	Rpia
O88983	Syntaxin-8	Stx8
Q99PL5	Ribosome-binding protein 1	Rrbp1
Q70IV5-2	Isoform 2 of Synemin	Synm
Q3UBX0	Transmembrane protein 109	Tmem109
P08122	Collagen alpha-2(IV) chain	Col4a2
O08734	Bcl-2 homologous antagonist/killer	Bak1
Q9JK37	Myozenin-1	Myoz1
Q9CQ89	Protein CutA	Cuta
Q9JJW5	Myozenin-2	Myoz2
P13541	Myosin-3	Myh3
O88990	Alpha-actinin-3	Actn3
Q8C0M9	Isoaspartyl peptidase 1/L-asparaginase	Asrgl1
O35658	Complement component 1 Q subcomponent-binding protein, mitochondrial	C1qbp
P20801	Troponin C2, skeletal muscle	Tnnc2
P00761	Trypsin	Trypsin
Q8R3G9	Tetraspanin-8	Tspan8
Q6NZB1	Protein arginine N-methyltransferase 6	Prmt6
P46412	Glutathione peroxidase 3	Gpx3
O70209	PDZ and LIM domain protein 3	Pdlim3
Q9ET78	Junctophilin-2	Jph2
O70251	Elongation factor 1-beta	Eef1b
Q5SX40	Myosin-1	Myh1
Q9Z2Y8	Pyridoxal phosphate homeostasis protein	Prosc
Q61292	Laminin subunit beta-2	Lamb2
Q5DTJ9	Myopalladin	Mypn
Q9D6J5	NADH dehydrogenase [ubiquinone] 1 beta subcomplex subunit 8, mitochondrial	Ndufb8
P16015	Carbonic anhydrase 3	Ca3
Q8BI84	Transport and Golgi organization protein 1 homolog	Mia3
O70373	Xin actin-binding repeat-containing protein 1	Xirp1
P08228	Superoxide dismutase [Cu-Zn]	Sod1
Q9QY76	Vesicle-associated membrane protein-associated protein B	Vapb
P02468	Laminin subunit gamma-1	Lamc1
Q9DCW4	Electron transfer flavoprotein subunit beta	Etfb
P51667	Myosin regulatory light chain 2, ventricular/cardiac muscle isoform	Myl2

Acession	Description	Gene
P97371	Proteasome activator complex subunit 1	Psme1
P13707	Glycerol-3-phosphate dehydrogenase [NAD(+)], cytoplasmic	Gpd1
Q922J3	CAP-Gly domain-containing linker protein 1	Clip1
O09044	Synaptosomal-associated protein 23	Snap23
Q9QZ23	NFU1 iron-sulfur cluster scaffold homolog, mitochondrial	Nfu1
P02535	Keratin, type I cytoskeletal 10	Krt10
Q9R0Y5-2	Isoform 2 of Adenylate kinase isoenzyme 1	Ak1
P63017	Heat shock cognate 71 kDa protein	Hspa8
P51885	Lumican	Lum
A2ASS6	Titin	Ttn
Q9DCB8	Iron-sulfur cluster assembly 2 homolog, mitochondrial	Isca2
Q9CZ13	Cytochrome b-c1 complex subunit 1, mitochondrial	Uqcrc1
Q8BJU0	Small glutamine-rich tetratricopeptide repeat-containing protein alpha	Sgta
O08795-2	Isoform 2 of Glucosidase 2 subunit beta	Prkcsh
Q3TVI8	Pre-B-cell leukemia transcription factor-interacting protein 1	Pbxip1
P00493	Hypoxanthine-guanine phosphoribosyltransferase	Hprt1
P68372	Tubulin beta-4B chain	Tubb4b
Q9Z2P8	Vesicle-associated membrane protein 5	Vamp5
P15532	Nucleoside diphosphate kinase 1	Nme1
P02469	Laminin subunit beta-1	Lamb1
P70296	Phosphatidylethanolamine-binding protein 1	Pebp1
P12787	Cytochrome c oxidase subunit 5A, mitochondrial	Cox5a
Q08376	Zinc finger and BTB domain-containing protein 14	Zbtb14
Q03265	ATP synthase subunit alpha 1, mitochondrial	Atp5a1
Q9CR00	26S proteasome non-ATPase regulatory subunit 9	Psmd9
P04370	Myelin basic protein	Mbp
O89116	Vesicle transport through interaction with t-SNAREs homolog 1A	Vti1a
P10637	Microtubule-associated protein tau	Mapt
P11531	Dystrophin	Dmd
Q9JKB1	Ubiquitin carboxyl-terminal hydrolase isozyme L3	Uchl3
Q5SX39	Myosin-4	Myh4
Q9Z172	Small ubiquitin-related modifier 3	Sumo3
Q62165	Dystroglycan 1	Dag1
O08532	Voltage-dependent calcium channel subunit alpha-2/delta-1	Cacna2d1
P26039	Talin-1	Tln1
O55103	Periaxin	Prx
Q91WP6	Serine protease inhibitor A3N	Serpina3n
Q9DCZ1	GMP reductase 1	Gmpr
P56480	ATP synthase subunit beta 5, mitochondrial	Atp5b
P09671	Superoxide dismutase 2 [Mn], mitochondrial	Sod2
O35381	Acidic leucine-rich nuclear phosphoprotein 32 family member A	Anp32a
Q66JS6	Eukaryotic translation initiation factor 3 subunit J-B	Eif3j2
Q64727	Vinculin	Vcl
Q9R1P0	Proteasome subunit alpha type-4	Psma4
Q9JIF9	Myotilin	Myot
Q99LM3	Smoothelin-like protein 1	Smtnl1
O35367	Keratocan	Kera
P02538	Keratin, type II cytoskeletal 6A	Krt6A
Q9WV55	Vesicle-associated membrane protein-associated protein A	Vapa
P08779	Keratin, type I cytoskeletal 16	Krt16
P07759	Serine protease inhibitor A3K	Serpina3k
P62979	Ubiquitin-40S ribosomal protein S27a	Rps27a
P09103	Protein disulfide-isomerase	P4hb

Acession	Description	Gene
P97300	Neuroplastin	Nptn
Q9CR68	Cytochrome b-c1 complex subunit Rieske, mitochondrial	Uqcrfs1
Q99M71	Mammalian ependymin-related protein 1	Epdr1
Q6P5H2	Nestin	Nes
Q9WTR5	Cadherin-13	Cdh13
Q7TQ48	Sarcalumenin	Srl
P50427	Steryl-sulfatase	Sts
Q9D1X0	Nucleolar protein 3	Nol3
P97457	Myosin regulatory light chain 2, skeletal muscle isoform	Mylpf
P07724	Serum albumin	Alb
P14602	Heat shock protein beta-1	Hspb1
P10518	Delta-aminolevulinic acid dehydratase	Alad
Q149B8	PGC-1 and ERR-induced regulator in muscle protein 1	Perm1
Q9D154	Leukocyte elastase inhibitor A	Serpinb1a
Q9CQ62	2,4-dienoyl-CoA reductase, mitochondrial	Decr1
Q6P9L6	Kinesin-like protein KIF15	Kif15
Q71LX4	Talin-2	Tln2
E9Q557	Desmoplakin	Dsp

Supplementary Table S3. Primers used in quantitative real-time polymerase chain reaction analysis.

Primers	Forward (5'-3')	Reverse (5'-3')
C1qbp	ACAGCATCCCTCCAACATTT	GGGAGTTGATGTCAGTTCTGG
Bax	TGTTTGCTGATGGCAACTTC	GATCAGCTCGGGCACTTTAG
Krt14	GCTCTTGTGGTATCGGTGGT	GAGGAGAAGCGAGAGGAGGT
Myh2	AGGCGGCTGAGGAGCACGTA	GCGGCACAAGCAGCGTTGG
Myh4	CAATCAGGAACCTTCGGAACAC	GTCCTGGCCTCTGAGAGCAT
Myh7	TGCCAAGGGCCTGAATGA	GCTTCCACCTAAAGGGCTGTT
Mef2c	GCAGGCAAAGATTGTGTGCT	CGCCTGTGTTACCTGCACTT
NFATc1	CCTCGAACCCTATCGAGTGT	GCCAGACAGCACCATCTTC
NFATc2	GAGCCCAGCGATGAGTATGA	CTGCTGTTGGTAGCGAGCAT
NFATc3	TTG TGT GAC CCA GCG TCA TT	CAT CAG ATC TGG AGA CGC CG
NFATc4	GAA GCT TGG GGC CAA CTC TA	AAC CTG TCC AGC CTG TTA GC
Atp2a2	GCCAGCTGTTCCACACTGTA	AAACATGCGCTGTGAGAAGC
Casq2	GCCCAGAGGACATGTTTGA	GATGTGGATCCCATTCAAGTC
Stab2	TTTGTGATGAAGGCATGGAA	CACAGGCTGTTCCACGAAA
Rn18s	CTCAACACGGGAAACCTCAC	CGCTCCACCAACTAAGAACG

Supplementary Table S4. Primers used in quantitative real-time polymerase chain reaction analysis.

Primers	Forward (5'-3')	Reverse (5'-3')
Casq2 promoter	GCTACCCGTCCTCTGTATGC	GGGCACCAAAAGTCAAAGAA
Atp2a2 promoter	AGGGCAACCAGCACAGAG	AGGGTGAGCCTGACTTTTCC
Stab2 promoter	CTGTCTTCCAAGGCAGGAAC	GCAGCCCTTTCCATACCATA

Figure S1

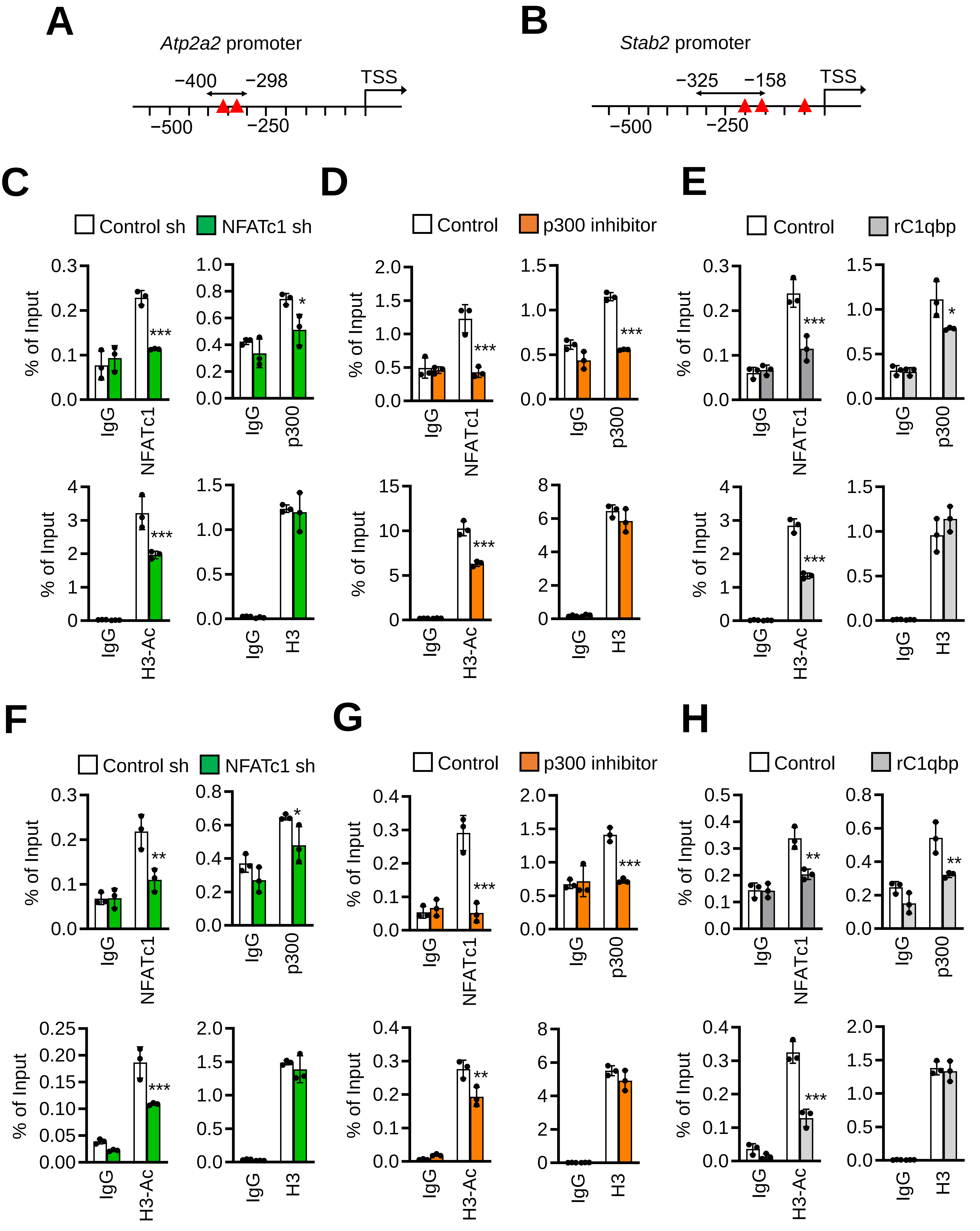

A
Atp2a2 promoter
−400
−298
TSS
−500
−250
B
Stab2 promoter
−325
−158
TSS
−500
−250
C
Control sh
NFATc1 sh
D
Control
p300 inhibitor
E
Control
rC1qbp
F
Control sh
NFATc1 sh
G
Control
p300 inhibitor
H
Control
rC1qbp
% of Input
IgG
NFATc1
p300
H3-Ac
H3

Fig. S1. Effects of NFATc1 knockdown, p300 inhibitor, and rC1qbp, on NFATc1/p300 localization and histone H3 acetylation at the *Atp2a2* and *Stab2* loci. (A and B) Schemes showing the *Atp2a2* (A) and *Stab2* (B) promoters with potential binding sites (red triangles) of NFATc1 predicted using the program PROMO (version 3.0.2; http://alggen.lsi.upc.es/cgi-bin/promo_v3/promo/promoinit.cgi?dirDB=TF_8.3). Arrows indicates the primers used for PCR in the chromatin immunoprecipitation (ChIP) assays. (C and F) C2C12 cells transduced with control shRNA or NFATc1 shRNA were differentiated in the presence of 2% horse serum for 5 days. ChIP assays for *Atp2a2* (C) and *Stab2* (F) were performed with anti-NFATc1, anti-p300, anti-acetylated histone H3 (H3-Ac), and anti-histone H3 (H3) antibodies. Data are represented as mean ± SD (n = 3). (D, E, G, and H) C2C12 cells pretreated with vehicle (control), rC1qbp (500 nM), or p300 inhibitor (20 μM) were differentiated in the presence of 2% horse serum for 5 days. ChIP assays for *Atp2a2* (D and E) and *Stab2* (G and H) were performed as described in (C and F). Data are represented as mean ± SD (n = 3). *$P < 0.05$;**$P < 0.01$; ***$P < 0.001$.

Figure S2

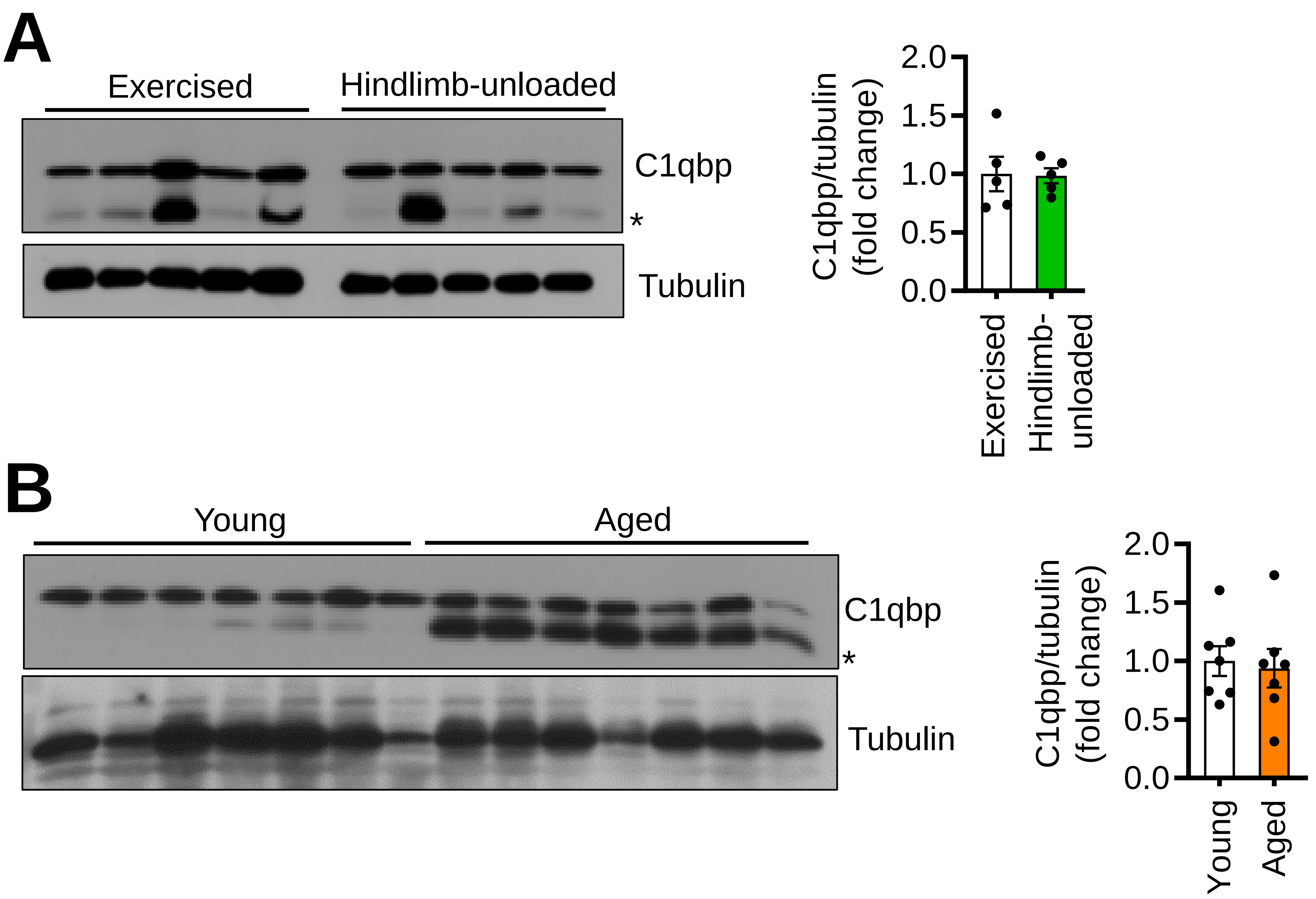

Fig. S2. Expression levels of C1qbp in mice with muscle loss. (A) Tibialis anterior muscles from exercised and hindlimb-unloaded male C57BL/6 mice were prepared. The expression level of C1qbp was analyzed using western blotting (left). The protein levels were quantified and normalized to tubulin levels (right). Data are represented as mean ± SEM (n = 5 mice). (B) Tibialis anterior muscles of 7-month-old (young) and 19-month-old (aged) male C57BL/6 mice were subjected to western blotting analysis (left). The quantification of the C1qbp protein levels is shown (right). Tubulin was used as a loading control. Data are represented as mean ± SEM (n = 7 mice). Asterisks indicate the non-specific bands. Original blots in (A,B) are presented in Supplementary Figure S8.

Figure S3

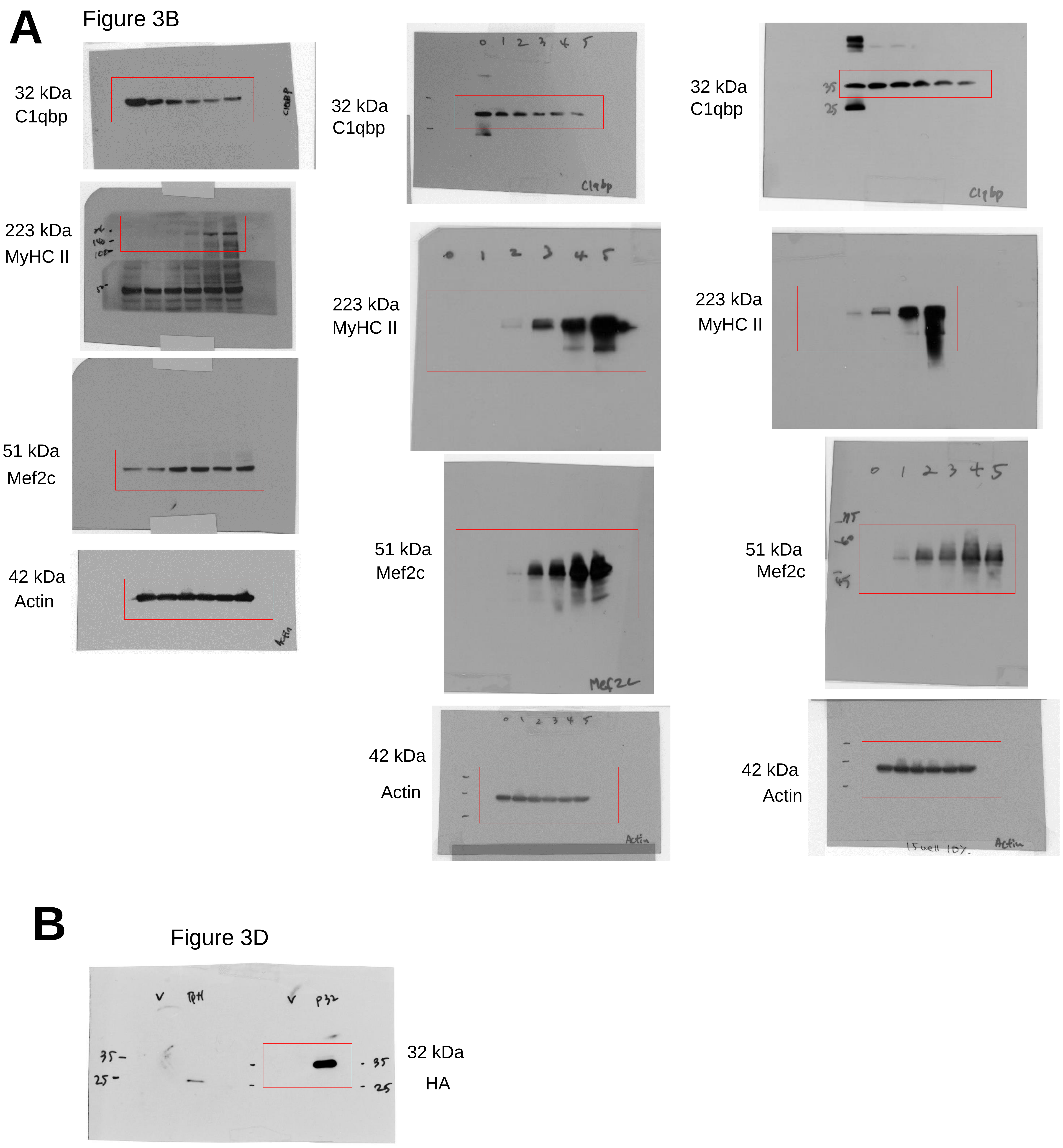

Fig. S3. Full-length and cutted membranes of all immunoblots related to Fig. 3. (A and B) Uncropped western blots are shown in A (Figure 3B) and B (Figure 3B). Molecular weight are indicated on the left or right side. Red rectangle represented the cropped area. Some membranes were cut prior to hybridization with antibodies.

Figure S4

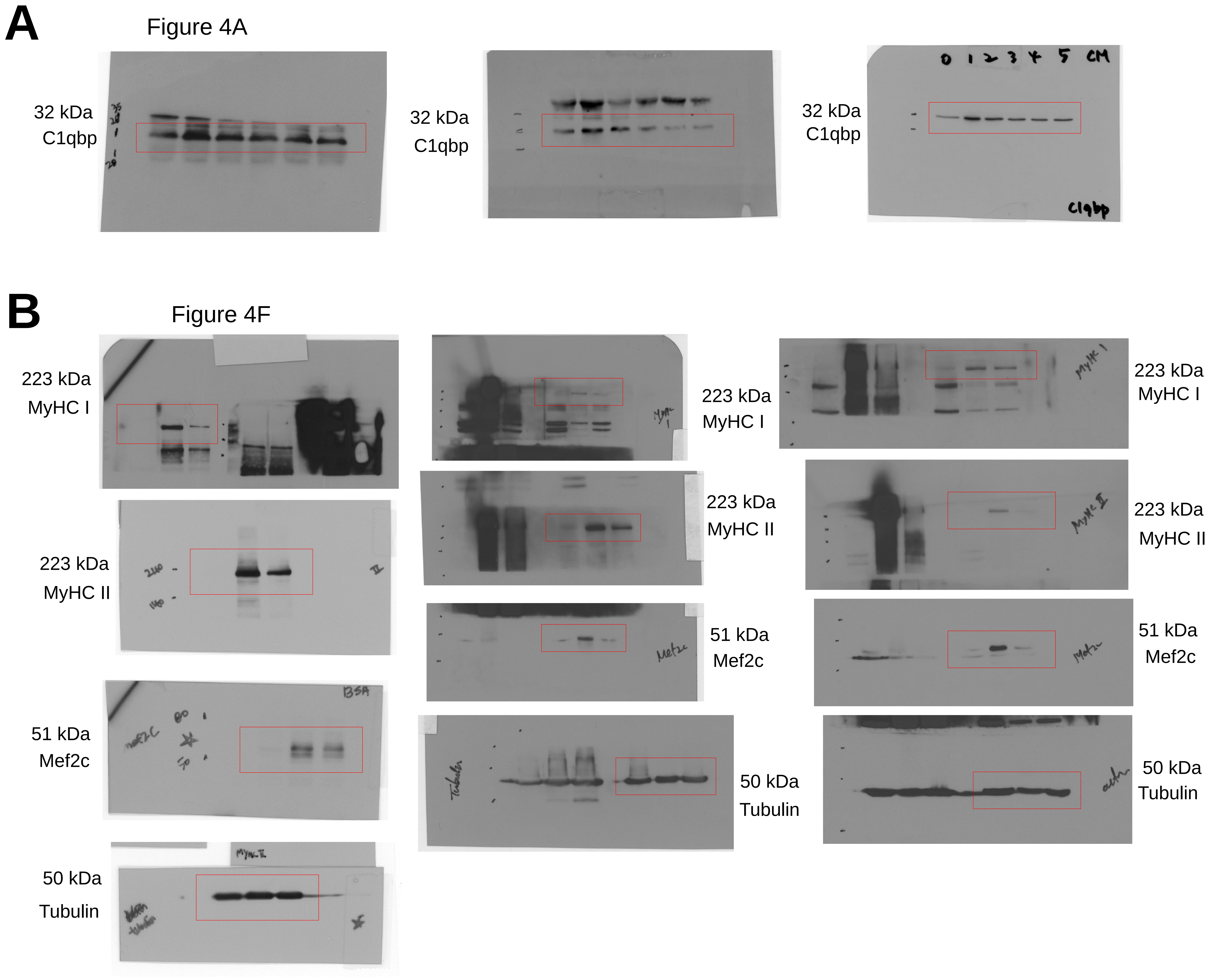

Fig. S4. Full-length and cutted membranes of all immunoblots related to Fig. 4. (A and B) Uncropped western blots are shown in A (Figure 4A) and B (Figure 4F). Molecular weight are indicated on the left or right side. Red rectangle represented the cropped area. Some membranes were cut prior to hybridization with antibodies.

Figure S5

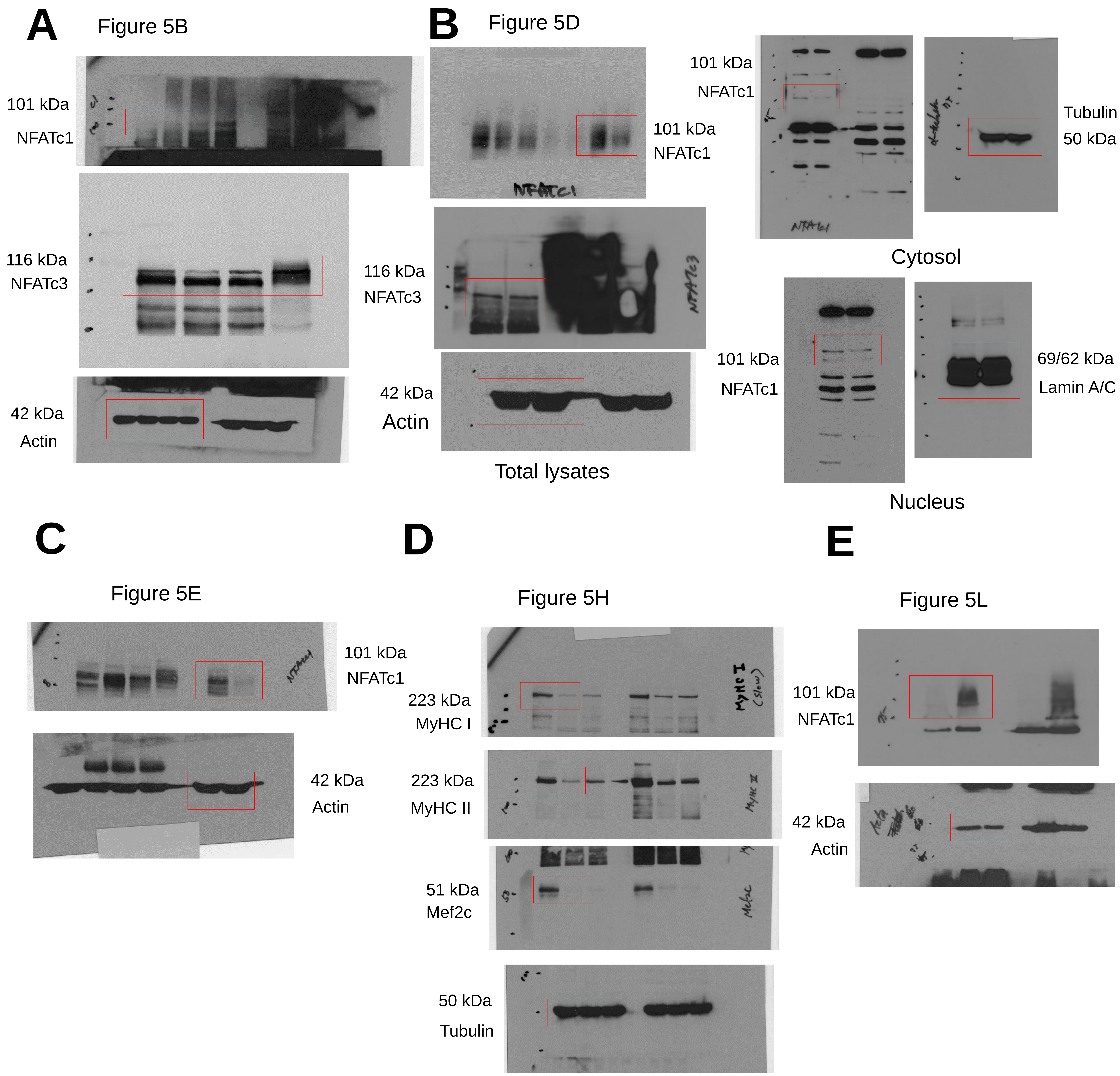

Fig. S5. Full-length and cutted membranes of all immunoblots related to Fig. 5. (A-E) Uncropped western blots are shown in A(Figure 5B) , B (Figure 5D) C (Figure 5E), D (Figure 5H), and E (Figure 5L). Molecular weight are indicated on the left or right side. Red rectangle represented the cropped area. Some membranes were cut prior to hybridization with antibodies.

Figure S6

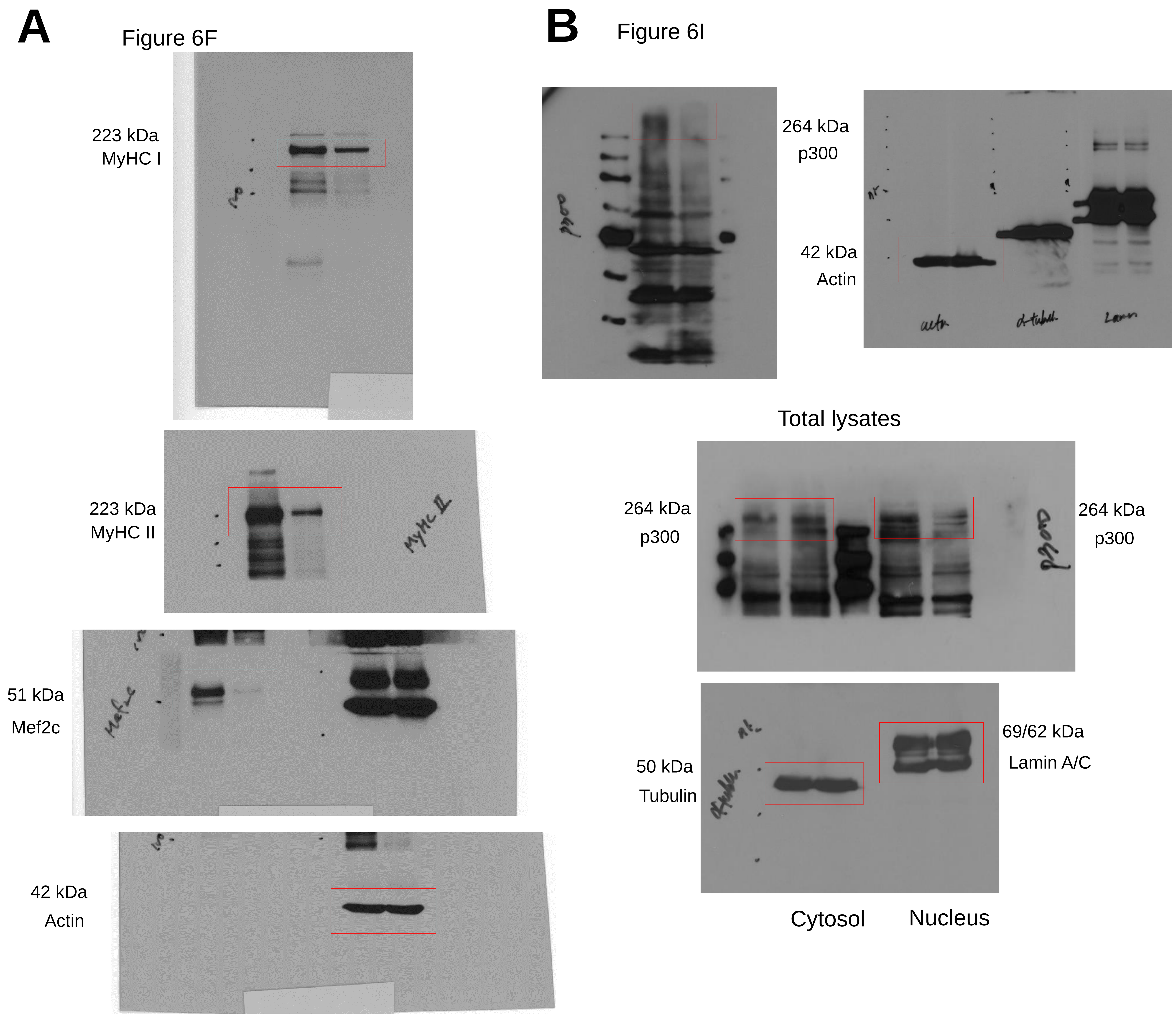

Fig. S6. Full-length and cutted membranes of all immunoblots related to Fig. 6. Uncropped western blots are shown in A (Figure 6F) and B (Figure 6I). Molecular weight are indicated on the left or right side. Red rectangle represented the cropped area. Some membranes were cut prior to hybridization with antibodies.

Figure S7

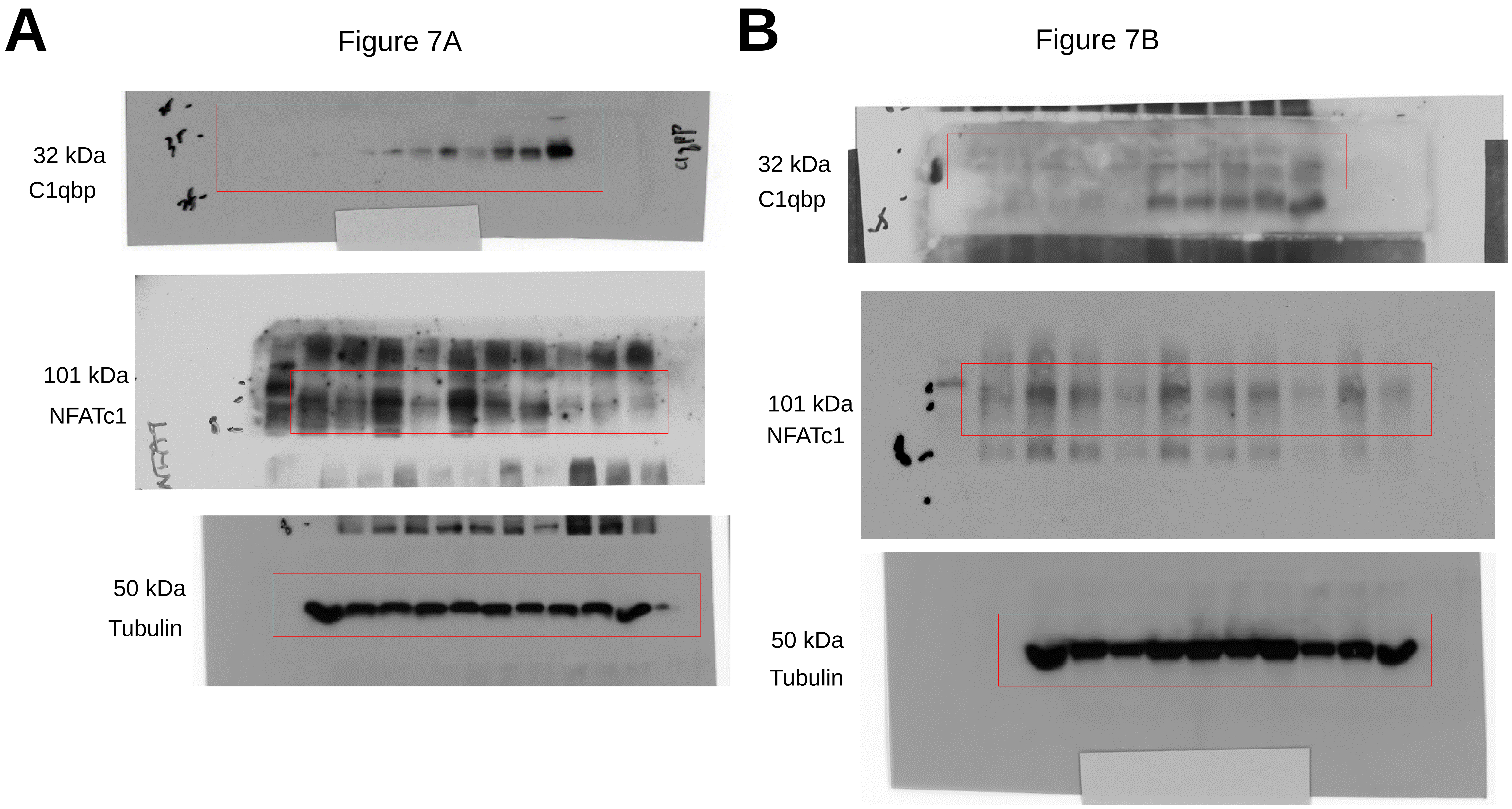

Fig. S7. Full-length and cutted membranes of all immunoblots related to Fig. 7. (A and B) Full-length and uncropped western blots are shown in A (Figure 7A) and B (Figure 7B). Molecular weight are indicated on the left side. Red rectangle represented the cropped area. Some membranes were cut prior to hybridization with antibodies.

Figure S8

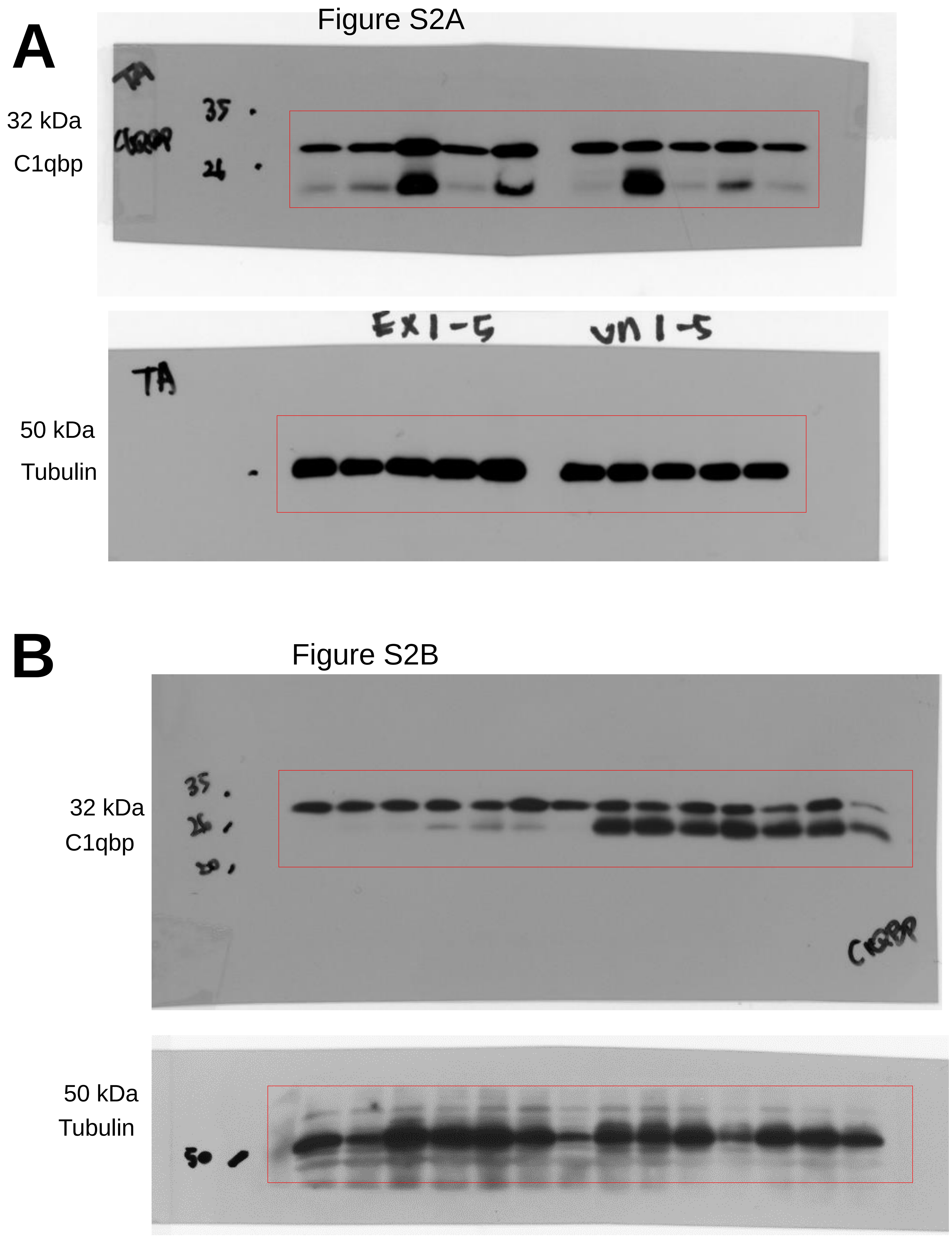

Fig. S8. Full-length and cutted membranes of all immunoblots related to Supplementary Fig. S2. (A and B) Full-length and uncropped western blots are shown in A (Figure S2A) and B (Figure S2B). Molecular weight are indicated on the left side. Red rectangle represented the cropped area. Some membranes were cut prior to hybridization with antibodies.
